# Antarctic Soil Metabolomics: A Pilot Study

**DOI:** 10.3390/ijms241512340

**Published:** 2023-08-02

**Authors:** Carlotta Ciaramelli, Alessandro Palmioli, Maura Brioschi, Simona Viglio, Maura D’Amato, Paolo Iadarola, Solveig Tosi, Laura Zucconi, Cristina Airoldi

**Affiliations:** 1 Department of Biotechnology and Biosciences, University of Milano—Bicocca, P.zza della Scienza 2, 20126 Milano, Italy; carlotta.ciaramelli@unimib.it (C.C.); alessandro.palmioli@unimib.it (A.P.); maura.brioschi@unimib.it (M.B.); 2 Biochemistry Unit, Department of Molecular Medicine, University of Pavia, Via Forlanini 6, 27100 Pavia, Italy; simona.viglio@unipv.it (S.V.); maura.damato01@universitadipavia.it (M.D.); 3 Department of Biology and Biotechnologies “L. Spallanzani”, University of Pavia, Via Adolfo Ferrata 9, 27100 Pavia, Italy; paolo.iadarola@unipv.it; 4 Department of Earth and Environmental Sciences, University of Pavia, Via S. Epifanio 14, 27100 Pavia, Italy; solveig.tosi@unipv.it; 5 Department of Ecological and Biological Sciences, University of Tuscia, Largo dell’Università snc, 01100 Viterbo, Italy; zucconi@unitus.it

**Keywords:** Antarctica, NMR spectroscopy, mass spectrometry, metabolites

## Abstract

In Antarctica, ice-free areas can be found along the coast, on mountain peaks, and in the McMurdo Dry Valleys, where microorganisms well-adapted to harsh conditions can survive and reproduce. Metabolic analyses can shed light on the survival mechanisms of Antarctic soil communities from both coastal sites, under different plant coverage stages, and inner sites where slow-growing or dormant microorganisms, low water availability, salt accumulation, and a limited number of primary producers make metabolomic profiling difficult. Here, we report, for the first time, an efficient protocol for the extraction and the metabolic profiling of Antarctic soils based on the combination of NMR spectroscopy and mass spectrometry (MS). This approach was set up on samples harvested along different localities of Victoria Land, in continental Antarctica, devoid of or covered by differently developed biological crusts. NMR allowed for the identification of thirty metabolites (mainly sugars, amino acids, and organic acids) and the quantification of just over twenty of them. UPLC-MS analysis identified more than twenty other metabolites, in particular flavonoids, medium- and long-chain fatty acids, benzoic acid derivatives, anthracenes, and quinones. Our results highlighted the complementarity of the two analytical techniques. Moreover, we demonstrated that their combined use represents the “gold standard” for the qualitative and quantitative analysis of little-explored samples, such as those collected from Antarctic soils.

## 1. Introduction

Antarctica presents one of the most physically and chemically demanding environments on Earth for biological organisms. Exposed soils exist only in isolated areas that remain ice-free seasonally or permanently, such as coastal oases, islands, mountain peaks, scree slopes, cliffs, and ice-free valleys, like those of the McMurdo Dry Valleys of the Victoria Land region. In these areas, microorganisms endure multiple stresses, including freezing temperatures, frequent freeze–thaw cycles, low nutrient availability, severe drought, high incidence of solar and UV radiation during the austral summer, and locally high salinity. Despite these harsh conditions, Antarctic soils surprisingly harbour a diversity of microbes, constituting the majority of the biomass of all Antarctic terrestrial systems [1,2].

Data on the biodiversity of these communities are still rare, with particular reference to their structure and functional diversity, as well as their capability to adapt to biotic and abiotic factors. Maritime Antarctic soils have received more attention in previous years [3,4,5], while fewer and scattered studies focused on those of continental Antarctica [6,7,8], where analyses are furthermore hindered by low DNA quantities, especially for inland soils, and the difficulty of distinguishing active organisms from relic DNA [9]. However, despite the extraordinary ability of soil communities to withstand conditions considered incompatible with active life, they are still not well characterised in terms of their stress adaptations. The investigation of these soil microorganisms’ activities, the metabolic profiling of their secretome and, more generally, of the soils hosting them, play a pivotal role, providing insight into the metabolism of these communities and the organic resources they can exploit to survive.

Nuclear Magnetic Resonance (NMR) spectroscopy and Mass Spectrometry (MS) are the best-performing techniques available today for metabolomics studies, and their integration could help to achieve a more comprehensive view of the metabolomic phenotype. However, examples of the application of both techniques to analyse soils are very rare, and even lacking for Antarctic soils.

Liquid Chromatography coupled with the mass spectrometry approach (LC-MS) was applied to soil metabolic profiling to measure microbe–metabolite relationships in situ and to characterise their dynamic composition in soil biocrusts from a temperate region [10]. Withers et al. [11] evaluated the discriminatory power of soil metabolomics and its potential use as a soil quality indicator, comparing, by Gas Chromatography coupled to mass spectrometry (GC-MS), the content of nine different topsoils collected along an altitudinal primary productivity gradient. The authors demonstrated a correlation between the metabolomic profile and several environmental factors, including pH, land use, moisture, and salinity. A pyrolysis GC-MS approach has been also applied to compare the parent materials to soil organic matters for three Antarctic podzols [12]. 

In addition, Coleine et al. [13] and Fanelli et al. [14] reported two examples of MS-based analyses of microbial communities colonising Antarctic rocks, comparing the metabolic responses of Antarctic endolithic communities when dormant, and after reanimation by wetting, light, and optimal temperature. 

NMR spectroscopy allowed for metabolomic analysis of soils from agricultural systems or native soils. Through samples grinding and extraction with sonication, Rochfort et al. [15] demonstrated the possibility of breaking up cell walls and thus characterise both extracellular and intracellular components of soil. Moreover, the components of agricultural soils, where microbial communities were influenced by the application of anaerobic soil disinfestation (ASD), were investigated by Johnsa and coworkers [16] by aqueous metabolites’ extraction from soil samples and subsequent 1D and 2D NMR analysis. Soucémarianadin et al. [17] combined NMR spectroscopy and MS analysis to characterise boreal soils and litter. They exploited liquid state 2D NMR spectroscopy to analyse dimethyl sulfoxide (DMSO) extracts of soil organic matter (SOM), but also pellets of original soil samples by solid state ^13^C NMR. Finally, a study by Abakumov and coworkers [18,19,20] described the targeted analysis of humic acids of selected sub-Antarctic soils by solid-state CP/MAS ^13^C NMR and ESR spectroscopy.

To the best of our knowledge, no studies on the untargeted metabolomic characterization of Antarctic soils have been conducted so far; moreover, none of the previous studies ever reported the combined use of NMR spectroscopy and MS. As a result, the primary goal of this study was to develop a protocol for detecting the metabolic profile of Antarctic soils, even those that are extremely oligotrophic and dry, and for which DNA extraction protocols often fail to yield acceptable amounts. This new tool will help to deepen the understanding of Antarctic microbial community functioning across different environments.

With this aim, different protocols were tested on soil samples collected in both coastal and inner sites of Victoria Land (continental Antarctica), the former covered by differently developed biological soil crusts (BSCs), the latter lacking autotrophic coverage.

## 2. Results

### 2.1. Antarctic Soil Samples’ Selection

**Figure 1:**
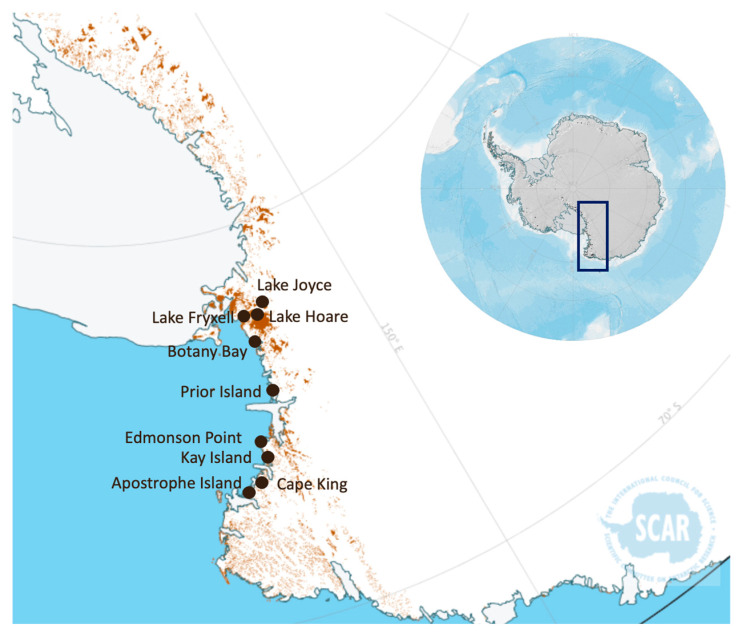
Sites of collection of the soil samples analysed in this pilot study. The blue rectangle indicates the Victoria’s Land sampling area. Map generated using the Antarctic Digital Database Map Viewer, https://www.add.scar.org/.

To set up an efficient protocol for the metabolomic analysis of Antarctic soils, we selected 20 samples among those collected within a previous study aimed at the microbiological characterization of soils along different localities of Victoria Land in continental Antarctica [21] (Figure 1).

**Figure 2:**
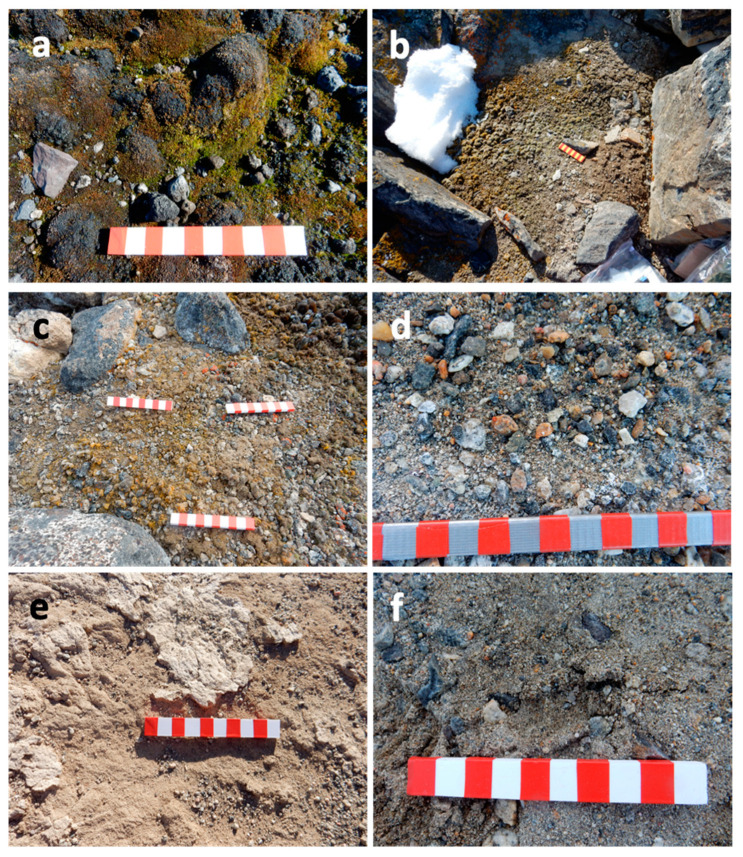
Examples of the different types of samples: (**a**) Edmonson Point; (**b**) Kay Island; (**c**) Apostrophe Island; (**d**) Lake Fryxell; (**e**) Lake Joyce; (**f**) Lake Hoare (Bars = 10 cm).

Thirteen samples of the twenty were from coastal localities, all in northern Victoria Land, except for Botany Bay, the southernmost one. All these soils were covered by differently developed biological crusts [21]. Among them, Edmonson Point and Botany Bay are Antarctic Specially Protected Areas (ASPAs; N. 154 and 165, respectively), harbouring exceptionally rich BSCs coverages. Seven samples were from inland localities, near to three lakes in the McMurdo Dry Valleys (South Victoria Land), namely Fryxell, Hoare, and Joyce, but far enough away from the lakes to have soils with low water activity and nutrient availability [21]. These samples were selected to test the efficiency of the metabolite extraction processes on both coastal samples, where a good recovery of metabolites was expected, and samples from Dry Valleys assumed to be very poor (Figure 2). 

The list of the samples and the types of superficial crusts are reported in Table 1. Soil physicochemical parameters are reported in Supplementary Material (Table S1).

### 2.2. Antarctic Soil Extract Preparation and NMR-Based Metabolic Profiling

**Figure 3:**
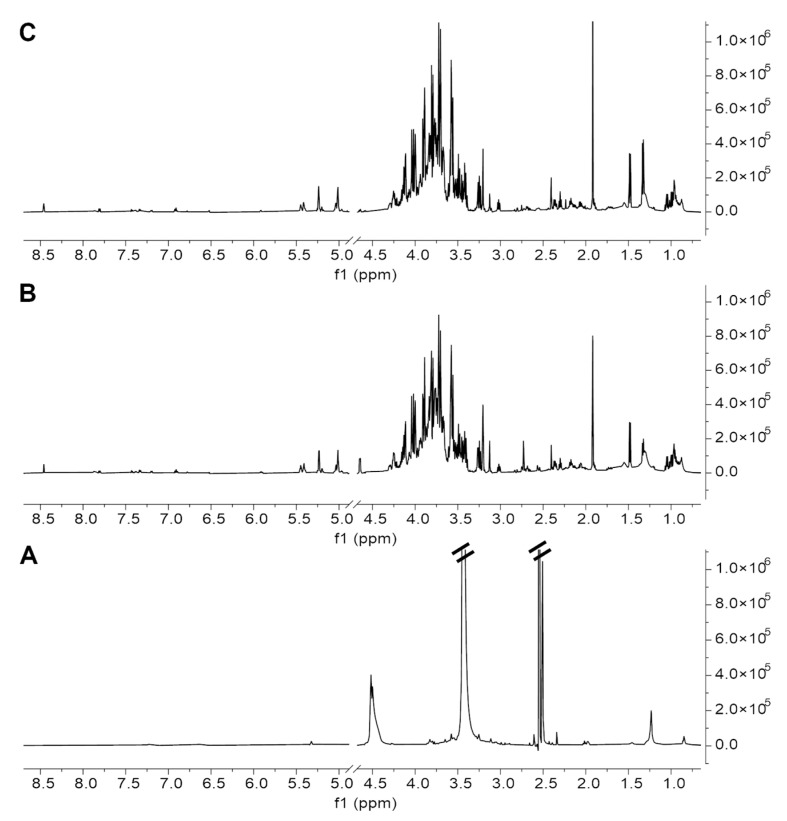
^1^HNMR spectra of extracts of Antarctic soils (Edmonson Point site 2) obtained by different extraction solvents: (**A**) DMSO; (**B**) H_2_O/MeOH (1:1), and (**C**) H_2_O/MeCN (7:3). Samples were dissolved at final concentration of 15 mg/mL in d_6_-DMSO (**A**) and in 10 mM phosphate buffer (PB) in D_2_O, pH 7.2 (**B**,**C**), with 0.5 mM TSP.

Soil extracts were prepared with ultrasound assisted extraction procedures with different solvents: dimethyl sulfoxide (DMSO), hydro-alcoholic extraction with water/methanol (1:1 *v*/*v*), and water/acetonitrile (7:3 *v*/*v*) (see Materials and Methods section for experimental details). For each procedure, the afforded ^1^H NMR metabolic profile of the extract was examined. Figure 3 shows, as an example, the comparison of the ^1^H NMR spectra acquired on the Edmonson Point (site 2) sample after extraction with the three solvents, clearly demonstrating that the extraction in DMSO was the least efficient, while the other two procedures afforded very similar spectra, both in terms of quality and quantity of the metabolites recovered (see also Supplementary Material—Table S2).

Given the substantial equivalence of the last two methods, and the extraction with water and acetonitrile (Figure 3C) also being fully compatible with the sample analysis by LC-MS, we selected the latter as the most suitable method to perform the metabolic profiling of Antarctic soils.

The ^1^H NMR spectra acquired on the twenty samples reported in Table 1 after extraction with water/acetonitrile (7:3) are depicted in Supplementary Material—Figure S1.

**Figure 4:**
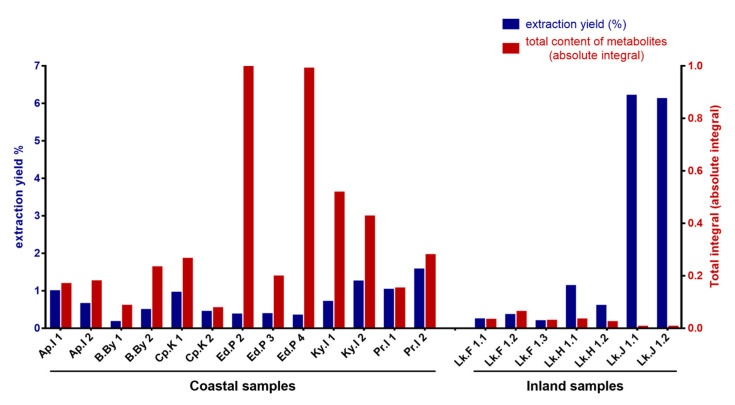
Comparison of the extraction yields (%) and the total content of metabolites determined by NMR as absolute integral, normalised to the integral with the highest value.

Figure 4 compares the total extraction yield afforded for each sample and the total integral of each corresponding ^1^H NMR spectrum, which can be considered, with a good approximation, as an estimation of the total content of the organic molecules (NMR-visible) contained in the extract. The graph clearly shows that the two parameters do not have a direct correlation. Notably, despite having the highest extraction yield, the couple of soil samples collected at Lake Joyce (Lk.J 1.1 and Lk.J 1.2) have an extremely low content of metabolites; this is an indication of the fact that almost all the material extracted from these samples is constituted by inorganic salts that do not have NMR-visible protons. On the other hand, despite the low total extraction yields, some other samples have higher metabolite contents, such as those from richly colonised soils collected at Edmonson Point (Ed.P 2 and Ed.P 4), which have the highest content of organic metabolites. Collectively, this analysis allows for a clustering of the coastal and inland samples, as can be easily deduced by observing the graph (Figure 4).

**Figure 5:**
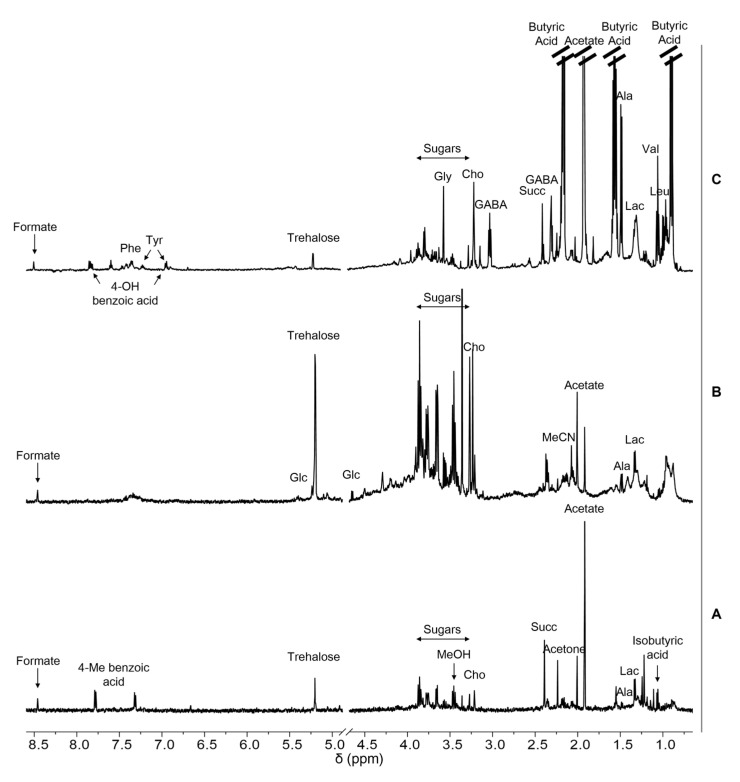
^1^H NMR spectra of extracts of Antarctic soils from different localities obtained in H_2_O/MeCN (7:3). (**A**) Lake Fryxell, (Lk.F 1.2); (**B**) Kay Island (Ky.I 2); (**C**) Edmonson Point (Ed.P 4). Samples are dissolved at final concentration of 15 mg/mL in 10 mM PB in D_2_O, pH 7.2, with 0.5 mM TSP. Assignments of the resonances of the most important metabolites are reported (Phe, phenylalanine; Tyr, tyrosine; Gly, glycine; Cho, choline; GABA, γ-aminobutyric acid; Succ, succinate; Ala, alanine; Lac, lactate; Val, valine; Leu, leucine; Glc, glucose).

Soil metabolites were identified through the analysis of 1D- (^1^H) (Figure 5 and Supplementary Material—Figure S1) and 2D- (^1^H,^1^H-TOCSY, ^1^H,^13^HHSQC) NMR spectra (Supplementary Material—Figures S2 and S3). 

Figure 5 shows the comparison of the ^1^H NMR spectra of three samples collected in different sites (Lake Fryxell, Lk.F 1.2; Kay Island, Ky.I 2; Edmonson Point, Ed.P 4) showing the assignment of the main metabolites. They were selected as representative of samples with a low, medium or high metabolite content, respectively, according to Figure 4. Table 2 reports the chemical shift assignment of the proton signals of all the metabolites identified in the soil samples analysed.

Compounds were manually identified with the support of 2D spectra (Supplementary Material—Figures S2 and S3) on a small pool of spectra. Then, a procedure already developed by our group for the analysis of metabolite mixtures from different types of samples was applied [22,23,24,25,26,27,28], in order to obtain the fast and semi-automatic identification and quantification of the metabolites present in all the Antarctic soil samples under examination. To this aim, a specific library, available as an .exp file [29], was created using the Simple Mixture Analysis (SMA) tool implemented in MestreNova 14.3.0-30573 software. An example of SMA output is depicted in Supplementary Material—Figure S4.

The concentrations (as μg/mg of extract) of the metabolites identified in all the Antarctic soil extracts are reported in Table 3.

### 2.3. UPLC/HR-MS Metabolic Profiling of Selected Soil Extracts

Due to the complementarity of the NMR-based and MS-based metabolomics approaches, extracts obtained from the samples Lk.F 1.2 (Lake Fryxell), Ky.I 2 (Kay Island), and Ed.P 4 (Edmonson Point) were simultaneously submitted also to Ultra-Performance Liquid Chromatography separation coupled with High Resolution Mass Spectrometry (UPLC/HR-MS) analysis. An untargeted approach was applied to obtain a comprehensive profiling of the selected soil extracts exploiting the data-independent acquisition method (MS^E^) under both negative and positive electrospray ionisation. First, samples were analysed as obtained from the reported extraction procedure, without any further treatment. Under this condition, a marked matrix effect emerged, resulting in a loss of response (ionisation suppression), reasonably due the high salinity of samples. To reduce the matrix effect, we decided to take advantage of sample dilution over alternative invasive or time-consuming cleanup approaches (e.g., SPE, QuEChERS) to rule out potential analyte losses [30]. We found that a five-fold dilution with blank solvent (aqueous 10% MeCN) slightly, but significantly, reduced the ionisation suppression due to matrix components, without affecting the detection limits. Spectrometric data were collected in triplicate for each sample and processed with MS-Dial 4.9, an open-source tool for compound identification in untargeted metabolomics [31]. Metabolite identification was achieved by considering accurate mass, isotopic pattern, and fragmentation pattern of each detected feature and through comparison with the spectral database. Overall, UPLC/HR-MS analysis allowed the identification of 23 compounds, and the determination of the molecular formula of four compounds whose structure remains unknown. Detailed spectrometric data were reported in Table 4 and measured fragmentation spectra versus matched reference spectra for all identified compounds were reported in Supplementary Material—pp. S10–S17.

## 3. Discussion

Metabolomics is one of the newly emerged fields of “omics” research aimed at the comprehensive analysis of the low-molecular-weight molecules (metabolites) present in biological systems. It can provide an overview of the metabolic processes and global biochemical events associated with a biological system, including the different communities that populate a particular environment. Indeed, recent studies have demonstrated the ability of soil metabolites to predict the presence/absence of taxa or the alteration of microbial mechanisms in response to climate changes, with the combination of metabolomic analysis (by MS or NMR) and communities’ characterization (by 16srRNA gene sequencing) [32,33]. Thus, metabolomics can help decipher the secrets that underlie the survival of metabolic active species in particularly inhospitable environments such as Antarctica. In this context, the best type of sample is represented by Antarctic soils that, especially in ice-free regions, host different communities of fungi and bacteria capable of adapting to extreme living conditions. Their slow growth or presence in a dormant state, the low water availability, the salt accumulation, and the limited number of primary producers make the setting of metabolomics of Antarctic soils particularly challenging; proof of this is the absence of previous studies reporting a detailed metabolomic analysis of the soils harvested in these regions.

In this study, therefore, we aimed at establishing extractive and analytical conditions allowing the metabolic profiling of both soils beneath well-developed BSCs and soils taken from regions where even the isolation of genetic material is difficult. We also combined the use of NMR spectroscopy and mass spectrometry to benefit from the main advantages of each of them and limit their specific disadvantages. For such a purpose, we extracted and analysed the metabolites from twenty different soil samples, showing crusts of different development stages and types, by comparing three different procedures, some reported in previous works [15,16,17] describing soil metabolomics.

The extraction procedure with water and acetonitrile was selected because of (1) the good extraction yield afforded for all the samples (Figure 4), (2) the broad NMR profile of metabolites (Figure 3), and (3) the minimal processing of extract samples required prior to being subjected to both NMR and MS analysis. In this context, the quantity and variety of metabolites observed by NMR spectroscopy represent the main discriminant, since, among the two analytical techniques, NMR is the most demanding, given the much lower sensitivity and the lack of coupling with a separation technique, which is the main advantage of MS. On the other hand, while the detection of a specific analyte in MS is strictly dependent on its chemistry and the chosen ionisation method [34,35], NMR is an intrinsically quantitative technique [36,37,38], and this not only allows to determine the absolute concentration of each metabolite above the limit of detection, but also to estimate the content of organic material (in terms of low molecular weight compounds) contained in a sample through the total integral value of the spectrum (Figure 4).

As already mentioned, the samples collected close to Lake Joyce (Lk.J 1.1 and Lk.J 1.2), affording the higher extraction yields, were among the poorest for content of organic material (Figure 4), as confirmed by NMR analysis. The same samples are among those presenting the highest cation exchange capacity (CEC), according to data reported in Supplementary Material (Table S1), suggesting that most of the extracted material consists of salt, with the content of organic material being extremely low. This highlights how the extraction yield in terms of *w*/*w* % on the amount of sample extracted is not a reliable parameter to estimate the efficiency of the extraction method when aimed at performing a metabolomics analysis.

NMR-based metabolic profiling allowed the identification of thirty metabolites and the quantification of just over twenty of them. They are mainly sugars, amino acids, and organic acids, both aliphatic and aromatic (Figure 5, Table 2 and Table 3). They are mostly metabolites characteristic of the major metabolic pathways common to every organism, some more characteristic of microorganism metabolism, such as trehalose, which are also implicated in the response to various environmental stresses [39].

Overall, these data are not indicative of the presence of particular species but, also given their quantitative nature, they allow us to understand how active the metabolism of the species hosted in the soil samples was and to make comparisons between soils.

Except for 4-hydroxybenzoic acid, these compounds were not detected through the UPLC/HR-MS analysis of the samples Ed.P 4, Ky.I 2, and Lk.F 1.2 selected as representative of high, medium and low content of organic material, respectively (Figure 4), but this is not surprising due to the characteristics of the metabolites identified by NMR. For example, carboxylic acids, such as acetate and butyrate, due to their strong hydrophilic properties, their poor ionisation efficiency, and their increased ion suppression in an electrospray ionisation (ESI) source, are difficult to separate and detect by LC-MS [40]. However, MS analysis allowed the identification of more than twenty other metabolites (Table 4 and Supplementary Material—S10–S17), not present in the NMR spectra, mainly belonging to the class of secondary metabolites, including flavonoids, medium- and long-chain fatty acids, benzoic acid derivatives, anthracenes, and quinones. Some of these metabolites can be directly traced back to the metabolism of plants, fungi, and lichens.

These results are not particularly surprising, as most of the metabolites identified by NMR are molecules with low volatility, which can be analysed by MS mainly after their derivatization [41,42]. From a practical point of view, however, this requires a further manipulation of the sample that the use of NMR can avoid. On the other hand, the compounds identified only by MS are NMR-visible molecules, but are evidently present in our samples at too low concentrations to allow their detection with this less sensitive technique.

## 4. Materials and Methods

### 4.1. Soil Sampling and Storage

Soil (0–5 cm depth) samples were aseptically collected during the XXXI Italian Antarctic Expeditions (December 2015–January 2016) by removing, where present, the superficial crust. They were stored at −20 °C until their analysis. An aliquot was used for metabolomic analyses, other two aliquots were previously used for physicochemical and molecular analyses.

### 4.2. Metabolomics Analyses

#### 4.2.1. Materials

All the solvents and reagents used for this work were purchased from Fisher Scientific unless indicated otherwise (Fisher Scientific International Inc., Pittsburgh, PA, USA). Reagent-grade water used to prepare all solutions was obtained from a Milli-Q (Millipore, Bedford, MA, USA) purification system.

#### 4.2.2. Soil Sample Extractions for NMR and MS Analyses

##### Extraction in DMSO

Antarctic soil (2 g) was suspended in 10 mL of DMSO, sonicated at 37 kHz, pulse, 100 pw, 30 min, 30–60 °C (Elmasonic P 30 H, Elma Schmidbauer GmbH, Singen, Germany). Each sample was centrifuged (Beckman Avanti J-20 Centrifuge, Beckman Coulter, Indianapolis, IN, USA) at 20,000× *g* for 15 min, 4 °C. The supernatant was collected and freeze-dried (Christ Alpha 1-2 LD plus, Martin Christ Gefriertrocknungsanlagen GmbH, Osterode am Harz, Germany). The extraction yield was calculated for each sample. Lyophilized samples were stored at −20 °C.

##### Extraction in H_2_O/CH_3_OH 1:1

Antarctic soil (2 g) was suspended in 10 mL of MilliQ water/CH_3_OH 1:1 with 50 µL NaN_3_ 0.3 M to obtain NaN_3_ 1.5 mM (0.01%), sonicated and centrifuged as described above. The supernatant was collected and CH_3_OH evaporated under reduced pressure at 40 °C (Heidolph Rotary Evaporator, Laborota 4000, Heidolph Instruments GmbH & Co. KG, Schwabach, Germany). The water phase was transferred in flask and freeze-dried as above. The extraction yield was calculated for each sample. Lyophilized samples were stored at −20 °C.

##### Extraction in H_2_O/CH_3_CN 7:3

Antarctic soil (2 g) was suspended in 10 mL of MilliQ water/CH_3_CN 7:3 with 50 µL NaN_3_ 0.3 M to obtain NaN_3_ 1.5 mM (0.01%) sonicated at 37 kHz, pulse, 100 pw, 60 min, 30–60 °C, and centrifuged as described above. The extraction was repeated with 10 mL of fresh extraction solution. The supernatants were transferred in a flask and freeze-dried as above. The extraction yield was calculated for each sample. Lyophilized samples were stored at −20 °C.

#### 4.2.3. NMR Metabolic Profiling of Soil Extracts

Freeze-dried samples of soil extracts were suspended in a 10 mM phosphate buffer (PB) in D_2_O or d_6_-DMSO at a final concentration of 15 mg/mL. Samples were sonicated (37 kHz, 10 min, Elmasonic P 30 H, Elma Schmidbauer GmbH, Singen, Germany) and centrifuged (12,000× *g*, 10 min, 4 °C, ScanSpeed 1730R Labogene, Lynge, Sweden) before NMR analyses. The 3-(trimethylsilyl)propionic-2,2,3,3-d_4_ acid sodium salt (TSP) was added to each sample at the final concentration of 0.5 mM, as an internal reference for concentrations and chemical shift. The pH of samples was verified with a microelectrode (InLab Micro electrode and Five Easy pHmeter, Mettler Toledo, Columbus, OH, USA) and adjusted to 7.2 with NaOD or DCl addition and corrected for the isotope effect.

NMR experiments were performed at 25 °C. All spectra were acquired on an AVANCE III 600 MHz NMR spectrometer (Bruker, Billerica, MA, USA). ^1^H NMR spectra were recorded with *zg* (^1^H in d_6_-DMSO) and *cpmgpr1d* (^1^H in D_2_O) pulse sequences in Bruker library and 256 scans, spectral width 20 ppm, relaxation delay 5 s. They were processed with 0.3 Hz line broadening, automatically phased and baseline corrected. Chemical shifts were internally calibrated to the TSP peak at 0.00 ppm. The ^1^H,^1^H TOCSY (Total Correlation Spectroscopy) spectra were acquired with 32 scans and 512 increments, 80 ms mixing time and relaxation delay 1.5 s. ^1^H,^13^C HSQC (Heteronuclear Single Quantum Coherence) spectra were acquired with 64 scans and 512 increments, relaxation delay 1.5 s.

MestReNova software package of Mestrelab (MestReNova v 14.3.0-30573, 13-06-2022, Mestrelab Research, Santiago de Compostela, Spain) was used for NMR spectra processing and peak picking.

The assignment of resonances and the identification of compounds were performed with the support of 2D NMR spectra, in-house libraries [43,44,45,46,47,48,49], the online databases Human Metabolome Database (HMDB, http://www.hmdb.ca), Biological Magnetic Resonance Data Bank (BMRB, http://www.bmrb.wisc.edu), FooDB (https://foodb.ca), Birmingham Metabolite Library (http://www.bml-nmr.org). All were accessed the last time on 1 July 2023.

The MNova Global Spectrum Deconvolution (GSD) algorithm was employed to deconvolute the overlapping regions, allowing the absolute quantification for metabolites with resonances in crowded spectral areas. The Simple Mixture Analysis (SMA) tool integrated in the MestreNova software package of Mestrelab (MestReNova v 14.3.0-30573, 13-06-2022, Mestrelab Research, Santiago de Compostela, Spain) was used to set a semi-automatic protocol for the identification and quantification of metabolites. A specific library for the matrix of interest was built and used in this work [29]. When possible, the concentration of the compound was calculated as the mean value of the different assigned signals [22,23,27,50,51].

#### 4.2.4. MS Metabolic Profiling of Selected Soil Extracts

High Resolution Mass Spectrometry (HRMS) analysis of selected soil extracts was performed using the ACQUITY UPLC H-class system coupled with the Xevo G2-XS QTof Mass Spectrometer (Waters Corp., Milford, MA, USA) through an ESI source. All the analytes were separated on the ACQUITY Premier HSS T3 Column (100 mm × 2.1 mm, 1.8 µm) coupled with VanGuard™ HSS T3 guard column (Waters Corp., Milford, MA, USA). The mobile phases were MS grade H_2_O (A) and MeCN (B), both containing 0.1% formic acid (HCOOH), and analyte elution was performed according to the following gradient: 0–1 min, 2% B; 1–11 min, 2–85% B linear gradient; 12–15 min isocratic 90% B; and then equilibrated for further 4 min at the initial conditions (2% B) before the next sample injection. Elution was performed at a flow rate of 0.4 mL/min, and the injection volume was 2 or 10 μL. The column temperature was set at 40 °C. Accurate mass data were collected both in positive and negative ionisation modes in a data independent manner, MS^E^, by alternating low and high energy applied to the collision cell. In the low energy MS mode, data were collected at constant collision energy of 6 eV; in high energy mode, the collision energy was ramped from 15 to 35 eV during each 0.1 s scan. Spectra were recorded in the range of m/z 50–1200. The source parameters were as follows: electrospray capillary voltage 1.5 kV, source temperature 140 °C, and desolvation temperature 600 °C. The cone and desolvation gas flows were 50 and 1000 L/h, respectively. The mass spectrometer was calibrated with 0.5 M sodium formate and leucine-enkephalin (200 pg/μL) infused at 8 μL/min and acquired every 30 sec was used as LockMass. MassLynx software (version 4.2) (Waters Corp., Milford, MA, USA) was used for instrument control, data acquisition, and data processing. MS Dial software version 4.9 was used for the peak picking, deconvolution, noise level setting, and identification of metabolites using the MS-DIAL metabolomics MSP spectral kit [31] (http://prime.psc.riken.jp/compms/msdial/main.html#MSP accessed on 1 July 2023).

## 5. Conclusions

The investigation of soil communities to deepen the understanding of Antarctic microbial community functioning across diverse environments requires the integration of different methodologies. Metabolomics of soils plays a pivotal role allowing to establish key biochemical processes exploited by Antarctic communities to adapt to these extreme conditions. However, no example of untargeted metabolomics analyses of Antarctic soils has been reported so far.

We therefore decided to fill this gap by developing an approach able to combine the use of NMR spectroscopy and MS, considered “the golden standard” for the analysis of organic compounds.

We optimized the procedure for metabolite extraction from soil samples and the conditions for the extracts analysis with both the analytical techniques. Results provided us with the qualitative and quantitative characterization of the metabolites contained in Antarctic soil samples. The different chemical nature of the identified molecules allows us to demonstrate the complementarity of the information provided by NMR and MS, not only justifying, but also prompting the combination of the use of the two techniques that allow obtaining a broader characterization of the metabolic profile of the samples under examination.

This feature appears fundamental when the metabolomics analysis of particularly poor and challenging samples, such as those from Antarctic soils, must be performed.

## Figures and Tables

**Table 1:** Soil samples analysed in this pilot study. CS = Coastal Site, IS = Inland Site (data from [21]).

Sample Code	Geographic Name	Coordinates	Type of Crust
Ap.I 1	Apostrophe Island site 1	CS—73°31′09.5″ S 167°25′55.3″ E—41 m asl	Grey crust with few mosses
Ap.I 2	Apostrophe Island site 2	CS—73°31′10.5″ S 167°25′55.3″ E—41 m asl	Brown thin crust with mosses and lichens
B.By 1	Botany Bay site 1	CS—77°00′26.0″ S 162°32′39.4″ E—115 m asl	Black thin crust disconnected from the below soil
B.By 2	Botany Bay site 2	CS—77°00′26.7″ S 162°32′40.5″ E—94 m asl	Black highly developed crust
Cp.K 1	Cape King site 1	CS—73°35′08.2″ S 166°37′19.2″ E—144 m asl	Black highly developed crust with mosses
Cp.K 2	Cape King site 2	CS—73°35′08.9″ S 166°37′3.5″ E—124 m asl	Dry thin and black crust with very few mosses
Ed.P 2	Edmonson Point site 2	CS—74°19′44.9″ S 165°07′39.9″ E—29 m asl	Thin signs of biological colonisation
Ed.P 3	Edmonson Point site 3	CS—74°19′45.2″ S 165°07′35.8″ E—31 m asl	Medium developed crust
Ed.P 4	Edmonson Point site 4	CS—74°19′45.1″ S 165°07′38.7″ E—30 m asl	Well-developed crust completely covered by mosses and lichens
Ky.I 1	Kay Island site 1	CS—74°04′12.6″ S 165°18′59.5″ E—190 m asl	Low developed crust with some mosses
Ky.I 2	Kay Island site 2	CS—74°04′11.8″ S 165°18′58.7″ E—61 m asl	Well-developed crust with more mosses
Pr.I 1	Prior Island site 1	CS—75°40′52.9″ S 162°53′38.3″ E—102 m asl	Grey thin and low developed crust
Pr.I 2	Prior Island site 2	CS—75°40′54.5″ S 162°53′45.8″ E—98 m asl	Wet highly developed crust dominated by mosses
Lk.F 1.1	Lake Fryxell site 1 sample 1	IS—77°36′7.2″ S 163°16′5.4″ E—28 m asl	Diffuse superficial crust, with saline efflorescence
Lk.F 1.2	Lake Fryxell site 1 sample 2	“	“
Lk.F 1.3	Lake Fryxell site 1 sample 3	“	“
Lk.H 1.1	Lake Hoare site 1 sample 1	IS—77°37′26.2″ S 162°53′27.8″ E—83 m asl	Low developed crust, with saline efflorescence
Lk.H 1.2	Lake Hoare site 1 sample 2	“	“
Lk.J 1.1	Lake Joyce site 1 sample 1	IS—77°42′21.1″ S 161°34′14.3″ E—448 m asl	Whitish consistent crusts on a sandy soil
Lk.J 1.2	Lake Joyce site 1 sample 2	“	“

**Table 2:** Assignments of ^1^H chemical shifts of the resonances of metabolites contained in extracts of Antarctic soils as found in ^1^H NMR and ^1^H,^1^H TOCSY spectra of samples dissolved in 10 mM PB in D_2_O, pH 7.2.

Metabolite	Assignment	^1^H Chemical Shift
Acetate	CH_3_	1.92 (s)
Acetone	2 × CH_3_	2.23 (s)
Acetonitrile	CH_3_	2.07 (s)
Alanine	CHα	3.79 (m)
	CH_3_	1.48 (d)
Aspartate	CHα	3.9 (m)
	CH_2_β	2.69 (m)–2.81 (dd)
Betaine	CH	3.89 (m)
	3 × CH_3_	3.25 (m)
Butyric acid	CH_2_ (1)	2.16 (t)
	CH_2_ (2)	1.56 (h)
	CH_3_	0.90 (t)
Choline	2 × CH_3_	3.21 (s)
Citrate	CH_2_ (2), CH_2_ (6)	2.65 (d)–2.56 (d)
Fatty acid	CH_2_	2.2 (t)
	CH_2_ (3)	1.55 (m)
	CH_2_ (ω2)	1.30 (m)
	CH_3_ (ω0)	0.90 (m)
Formate	O=C-H	8.46 (s)
GABA (γ-Aminobutyric acid)	CH (1)	3.00 (t)
	CH (3)	2.30 (t)
	CH (2)	1.89 (m)
α-D-Glucose	CH (1)	5.24 (d)
	CH (2)	3.85 (m)
	CH_2_ (6)	3.82–3.76 (m)
	CH (3)	3.72 (m)
	CH (5)	3.54 (m)
	CH (4)	3.42 (m)
β-D-Glucose	CH (1)	4.65 (d)
	CH_2_ (6)	3.71–3.89 (m)
	CH (3), CH (5)	3.47 (m)
	CH (4)	3.41 (m)
	CH (2)	3.24 (m)
Glutamate	CHα	3.76 (m)
	CH_2_γ	2.34 (m)
	CH2β	2.08 (m)
Glycine	CH_2_	3.56 (s)
4-Hydroxybenzoic acid	CH (2, 6)	7.81 (d)
	CH (3, 5)	6.91 (d)
Isobutyric acid	2 × CH_3_	1.06 (d)
	CH	2.39 (m)
Isoleucine	CHα	3.71 (d)
	CH	1.97 (m)
	CH_2_	1.25–1.46 (m)
	CH_3_ (7)	1.00 (d)
	CH_3_ (6)	0.91 (t)
Lactate	CH	4.11 (q)
	CH_3_	1.33 (d)
Leucine	CHα	3.72 (d)
	CH_2_ + CH	1.71 (m)
	2 × CH_3_	0.95 (t)
Malic acid	CH_2_ (3)	2.36 (dd)–2.67 (dd)
Methanol	CH_3_	3.36 (s)
4-Methylbenzoic acid	CH (2, 6)	7.78 (d)
	CH (3, 5)	7.32 (d)
	CH_3_	2.39 (s)
Phenylalanine	CH (7), CH (9)	7.43 (d)
	CH (8)	7.38 (m)
	CH (6), CH (10)	7.32 (d)
	CHα	3.98 (m)
	CH2β	3.10 (dd)–3.27 (dd)
Succinate	2 × CH_2_	2.39 (s)
Sucrose	CH (7) (Glc)	5.42 (d)
	CH (3) (Fru)	4.22 (d)
	CH (4) (Fru)	4.06 (t)
	CH (5) (Fru)	3.88 (m)
	CH (9) (Glc)	3.83 (m)
	CH 17+19	3.81 (m)
	CH (11) (Glc)	3.75 (t)
	CH (13) (Fru)	3.67 (s)
	CH (12) (Glc)	3.56 (dd)
	CH 1(0) (Glc)	3.47 (t)
Trehalose	CH (1)	5.20 (d)
	CH_2_ (6)	3.85 (m)
	CH (3), CH (5)	3.77 (m)
	CH (4)	3.66 (dd)
	CH (2)	3.46 (t)
Tyrosine	CH (7), CH (10)	7.20 (d)
	CH (6), CH (11)	6.90 (d)
	CHα	3.92 (m)
	CH_2_β	3.04 (dd)–3.18 (dd)
Uridine	CH (6)	7.86 (m)
	CH (5) pyrimidine + CH (1′) ribose	5.92 (m)
	CH (2′) Ribose	4.36 (m)
	CH (3′) Ribose	4.24 (m)
	CH (4′) Ribose	4.12 (m)
Valine	CHα	3.60 (d)
	CH (2)	2.26 (m)
	CH_3_ (6)	1.04 (d)
	CH_3_ (3)	0.98 (d)

**Table 3:** Quantitation of metabolites contained in extracts of Antarctic soils as found in ^1^H NMR spectra of samples dissolved in 10 mM PB in D_2_O, pH 7.2. Metabolites were quantified using the SMA tool of MestreNova software (MestReNova v 14.3.0-30573, 13-06-2022, Mestrelab Research, Santiago de Composte-la, Spain) and reported as μg of metabolite/mg of extract. ND (not detectable) is due to signal overlapping or signals with too low intensity.

	**Acetate**	**Acetone**	**Alanine**	**Aspartate**	**Butyric Acid**		**Choline**	**Formate**	**GABA**	**Glucose**	**Glycine**
Ap.I 1	11.89	2.47	ND	ND	ND		2.17	2.37	ND	ND	ND
Ap.I 2	7.05	4.07	ND	ND	ND		1.47	2.23	ND	ND	ND
B.By 1	11.77	1.48	ND	ND	ND		2.08	4.88	ND	ND	ND
B.By 2	12.73	3.32	7.13	ND	ND		8.26	ND	ND	80.47	ND
Cp.K 1	10.49	3.74	12.35	ND	ND		3.34	ND	ND	ND	ND
Cp.K 2	8.45	1.39	ND	ND	ND		0.77	1.10	ND	ND	ND
Ed.P 2	57.61	8.87	47.57	10.20	ND		16.11	6.66	7.84	391.54	ND
Ed.P 3	91.08	1.00	10.69	ND	65.44		1.78	1.55	12.58	ND	5.10
Ed.P 4	130.11	1.96	19.46	ND	98.98		4.26	1.08	19.10	ND	6.21
Ky.I 1	76.82	6.54	56.36	15.00	ND		ND	11.81	8.87	191.57	ND
Ky.I 2	13.17	4.03	14.91	ND	ND		7.15	5.06	ND	159.86	17.32
Pr.I 1	5.56	1.78	4.99	ND	ND		4.55	2.78	ND	ND	7.76
Pr.I 2	164.50	3.71	16.10	ND	ND		4.99	ND	21.66	73.50	14.81
Lk.F 1.1	6.21	ND	ND	ND	ND		2.51	ND	ND	ND	ND
Lk.F 1.2	11.17	ND	ND	ND	ND		1.10	1.99	ND	ND	2.67
Lk.F 1.3	5.36	0.73	ND	ND	ND		0.40	1.11	ND	ND	ND
Lk.H 1.1	6.41	1.73	ND	ND	ND		3.36	ND	ND	ND	ND
Lk.H 1.2	4.48	1.70	ND	ND	ND		2.68	ND	ND	ND	ND
Lk.J 1.1	ND	ND	ND	ND	ND		ND	ND	ND	ND	ND
Lk.J 1.2	0.83	0.98	ND	ND	ND		ND	ND	ND	ND	ND
	**4-Hydroxybenzoic Acid**	**Isobutyric Acid**	**Lactate**	**Methanol**	**4-Methylbenzoic Acid**	**Phenylalanine**	**Succinate**	**Sucrose**	**Trehalose**	**Tyrosine**	**Valine**
Ap.I 1	ND	2.01	15.19	1.04	10.98	ND	7.66	ND	73.02	ND	ND
Ap.I 2	ND	ND	16.45	3.05	ND	ND	ND	30.12	109.76	ND	ND
B.By 1	ND	ND	10.33	7.05	ND	ND	1.53	ND	46.32	ND	ND
B.By 2	ND	1.57	13.87	1.29	12.25	ND	10.55	99.95	221.58	ND	ND
Cp.K 1	ND	1.42	34.71	2.35	ND	ND	ND	ND	113.41	ND	ND
Cp.K 2	ND	1.30	8.77	0.38	7.13	ND	4.87	ND	25.56	ND	ND
Ed.P 2	10.50	ND	86.18	ND	ND	24.23	ND	138.75	114.56	11.54	29.52
Ed.P 3	0.93	0.55	9.25	0.30	ND	ND	1.08	ND	ND	ND	ND
Ed.P 4	2.38	2.54	13.92	0.21	ND	8.33	2.58	7.57	20.50	4.07	ND
Ky.I 1	ND	ND	68.40	3.57	ND	83.92	ND	133.95	153.12	35.15	59.98
Ky.I 2	ND	ND	34.05	15.40	ND	ND	ND	ND	319.02	ND	ND
Pr.I 1	ND	ND	15.79	12.69	ND	ND	ND	49.75	43.81	ND	ND
Pr.I 2	ND	5.73	16.51	1.29	ND	28.74	4.82	ND	92.65	ND	ND
Lk.F 1.1	ND	ND	3.07	5.68	ND	ND	3.21	ND	15.38	ND	ND
Lk.F 1.2	ND	1.67	10.69	1.49	12.71	ND	11.65	ND	39.48	ND	ND
Lk.F 1.3	ND	ND	4.81	6.28	ND	ND	1.76	ND	12.37	ND	ND
Lk.H 1.1	ND	ND	3.77	5.87	ND	ND	1.36	ND	17.34	ND	ND
Lk.H 1.2	ND	ND	5.69	0.07	4.43	ND	3.78	ND	8.63	ND	ND
Lk.J 1.1	ND	ND	ND	0.53	ND	ND	ND	ND	ND	ND	ND
Lk.J 1.2	ND	ND	ND	ND	ND	ND	ND	ND	ND	ND	ND

**Table 4:** Detailed UPLC/HR-MS data for the main components identified in extracts of Antarctic soil samples from Edmonson Point (Ed.P 4), Kay Island (Ky.I 2) and Lake Fryxell (Lk.F 1.2).

ID	Rt (min)	Average (m/z)	Metabolite Name	Adduct Type	Reference (m/z)	Error (ppm)	Formula	Ontology	INCHIKEY	Sample	Possible Sources ^a^
1	2.09	268.1044	Adenosine	[M+H]^+^	268.1046	−0.45	C_10_H_13_N_5_O_4_	Purine nucleosides	OIRDTQYFTABQOQ-KQYNXXCUSA-N	Ed.P, Ky.I	Ubiquitous
2	3.20	120.0812	Phenylethanolamine	[M−H_2_O+H]^+^	120.0808	3.53	C_8_H_11_NO	Aralkylamines	ULSIYEODSMZIPX-UHFFFAOYSA-N	Ed.P, Ky.I	Ubiquitous
3	4.00	181.0502	4-Hydroxyphenyllactic acid	[M−H]^−^	181.0506	−2.39	C_9_H_10_O_4_	Benzenoids	HXIPUYVSSGKLFF-UHFFFAOYSA-N	Ed.P	Microbes
4	4.27	137.0244	4-Hydroxybenzoic acid	[MH]^−^	137.0244	0.00	C_7_H_6_O_3_	Hydroxybenzoic acid derivatives	FJKROLUGYXJWQN-UHFFFAOYSA-N	Ed.P	Plants and microbes
5	4.31	609.1466	Luteolin 6-C-glucoside 8-C-arabinoside	[M−H]^−^	609.1461	0.71	C_27_H_30_O_16_	Flavonoid 8-C-glycosides	ZLPSOQFIIQIIAX-UHFFFAOYNA-N	Ed.P	Plants
6	4.51	159.0664	3-Methyladipic acid	[M−H]^−^	159.0663	0.31	C_7_H_12_O_4_	Medium-chain fatty acids	SYEOWUNSTUDKGM-UHFFFAOYSA-N	Ed.P	Animals
7	4.53	593.1517	Vicenin 2	[M−H]^−^	593.1512	0.93	C_27_H_30_O_15_	Flavonoid 8-C-glycosides	FIAAVMJLAGNUKW-UHFFFAOYSA-N	Ed.P	Plants
		595.1667		[M−H]^+^	595.1658	1.55					
8	4.62	377.14664	(-)-Riboflavin	[M+H]^+^	377.1461	1.46	C_17_H_20_N_4_O_6_	Flavins	AUNGANRZJHBGPY-SCRDCRAPSA-N	Ed.P, Ky.I	Ubiquitous
9	4.74	213.9642	2-Benzothiazolesulfonic acid	[M−H]^−^	213.9638	1.82	C_7_H_5_NO_3_S_2_	Benzothiazoles	ZCXGMSGCBDSEOY-UHFFFAOYSA-N	Ed.P, Lk.F	Contaminant
		215.9790		[M+H]^+^	215.9784	2.92					
10	5.22	173.0816	Suberic acid	[M−H]^−^	173.0819	−1.91	C_8_H_14_O_4_	Medium-chain fatty acids	TYFQFVWCELRYAO-UHFFFAOYSA-N	Ed.P, Ky.I	-
11	5.84	271.1550	C_14_H_2_4O_5_	[M−H]^−^	271.1551	−0.37	C_14_H_24_O_5_	TBD ^b^	TBD ^b^	Ed.P	-
12	6.54	236.0973	C_10_H_15_N_5_S	[M−H]^−^	236.0975	−0.85	C_10_H_15_N_5_S	TBD ^b^	TBD ^b^	Ed.P, Lk.F	-
13	6.84	421.1860	C_22_H_30_O_8_	[M−H]^−^	421.1868	−1.90	C_22_H_30_O_8_	TBD ^b^	TBD ^b^	Ed.P, Lk.F	-
14	7.11	195.0300	Haematommic acid	[M−H]^−^	195.0299	0.43	C_9_H_8_O_5_	Hydroxybenzoic acids	KCOOTJRKKIDHTM-UHFFFAOYSA-N	Ky.I	Lichens: *Asahinea chrysantha*
15	7.19	269.0452	Apigenin	[M−H]^−^	269.0450	0.82	C_15_H_10_O_5_	Flavones	KZNIFHPLKGYRTM-UHFFFAOYSA-N	Ed.P, Ky.I	Plants
		271.0608		[M+H]^+^	271.0601	2.58					
16	7.37	299.0564	Kaempferide	[M−H]^−^	299.0561	0.94	C_16_H_12_O_6_	Flavonols	SQFSKOYWJBQGKQ-UHFFFAOYSA-N	Ed.P	Plants
		301.0712		[M+H]^+^	301.0712	0.10					
17	7.51	237.1493	C_14_H_20_O_3_	[M+H]^+^	237.1485	3.37	C_14_H_20_O_3_	TBD ^b^	TBD ^b^	Ed.P	-
		235.1342		[M−H]^−^	235.1340	0.85					
18	7.69	299.0194	Emodic acid	[M−H]^−^	299.0197	0.95	C_15_H_8_O_7_	Anthracenecarboxylic acids	ZJXVNNSMRGTDBI-UHFFFAOYSA-N	Ky.I	Lichens and Fungi
19	8.15	323.0557	Sterigmatocystin	[M−H]^−^	323.0561	−1.24	C_18_H_12_O_6_	Sterigmatocystins	UTSVPXMQSFGQTM-UHFFFAOYNA-N	Ky.I	Fungi: *Aspergillus*
20	8.47	371.0401	Norstictic acid	[M−H]^−^	341.0409	−2.03	C_18_H_12_O_9_	Diarylethers	IEVVSJFLBYOUCJ-UHFFFAOYSA-N	Ky.I	Lichens: *Buellia, Dimelaena, Usnea*
21	9.02	313.0352	Parietinic acid	[M−H]^−^	313.0350	0.61	C_16_H_10_O_7_	Anthracenecarboxylic acids	HEULMVKOOVHXME-UHFFFAOYSA-N	Ky.I	Lichens: *Xanthoria, Fulgensia*
22	9.04	307.0613	Pulvinic acid	[M−H]^−^	307.0610	0.81	C_18_H_12_O_5_	Butenolides	CMFBGFRHPQTELQ-JQIJEIRASA-N	Ky.I	Lichens: *Letharia, Candelaria.* Fungi: *Aspergillus*
23	9.09	305.0448	Calycin	[M−H]^−^	305.0450	−0.69	C_18_H_10_O_5_	Benzofurans	CGRCGRBHNKRILW-JQIJEIRASA-N	Ky.I	Lichens: *Candelaria, Pseudocyphellaria*
24	9.70	269.0454	Emodin	[M−H]^−^	269.0455	−0.22	C_15_H_10_O_5_	Hydroxyanthraquinones	RHMXXJGYXNZAPX-UHFFFAOYSA-N	Ky.I	Plants, Fungi and crustose lichen
25	9.71	275.2009	(9S,13S)-12-Oxophytodienoate	[M−H_2_O+H]^+^	275.2006	1.20	C_18_H_28_O_3_	Prostaglandins and related compounds	PMTMAFAPLCGXGK-TTXFDSJOSA-N	Ed.P	Plants
		293.2119		[M+H]^+^	293.2111	2.66					
26	11.11	297.2426	Lichesterylic acid	[M−H]^−^	297.2435	−3.16	C_18_H_34_O_3_	Long-chain fatty acids	FYKXUGIGKHKTDH-UHFFFAOYSA-N	Ed.P	Lichens
27	11.17	374.9593	6-O-Methylarthothelin	[M+H]^+^	374.9580	3.49	C_15_H_9_Cl_3_O_5_	Xanthones	SMLUHOHPDVBXKH-UHFFFAOYSA-N	Ky.I	Lichen: *Lecanora hybocarpa, Dimelaena*
		372.9445		[M−H]^−^	372.9440	1.31					

^a^ source: LOTUS https://lotus.naturalproducts.net/ accessed on 1 July 2023, PUBCHEM https://pubchem.ncbi.nlm.nih.gov/ accessed on 1 July 2023; ^b^ TBD: to be determined.

## Data Availability

The specific SMA library for the identification and quantification of metabolites present in NMR spectra is available [29]. https://board.unimib.it/datasets/h2pbhgv9wc/1 https://board.unimib.it/datasets/h2pbhgv9wc/1.

## Supplementary Materials

The following supporting information can be downloaded at: https://www.mdpi.com/article/10.3390/ijms241512340/s1.
