# Supplementary material for: Antarctic Soil Metabolomics: A Pilot Study
Source: Int J Mol Sci. 2023 Aug 2;24(15):12340. doi: 10.3390/ijms241512340 (PMC10418359; doi:10.3390/ijms241512340)
Supplement: Supplementary file 1 [file ijms-24-12340-s001.zip › ijms-2521071-supplementary.pdf]

ANTARCTIC SOIL METABOLOMICS: A PILOT STUDY

Carlotta Ciaramelli 1,†, Alessandro Palmioli 1,†, Maura Brioschi 1, Simona Viglio 2, Maura D'Amato 2, Paolo Iadarola 3, Solveig Tosi 4, Laura Zucconi 5 and Cristina Airoldi 1,*

1. Department of Biotechnology and Biosciences, University of Milano—Bicocca, P.zza della Scienza 2, 20126 Milano, Italy.

2. Biochemistry Unit, Department of Molecular Medicine, University of Pavia, Via Forlanini 6, 27100 Pavia, Italy.

3 Department of Biology and Biotechnologies "L. Spallanzani", University of Pavia, Via Adolfo Ferrata 9, 27100 Pavia, Italy.

4 Department of Earth and Environmental Sciences, University of Pavia, via S. Epifanio 14, 27100 Pavia, Italy.

5 Department of Ecological and Biological Sciences, University of Tuscia, Largo dell'Università snc, 01100 Viterbo, Italy.

***** Correspondence: cristina.airoldi@unimib.it; Tel.: +39-0264483303

† These authors contributed equally to this work.

Supplementary Materials

Table of contents

Table S1 p. S2

Table S2 p. S3-S5

Figure S1 p. S6

Figure S2 p. S7

Figure S3. p. S8

Figure S3 p. S9

MS/MS spectra of identified compounds p. S10-S17

Table S1. Physicochemical parameters of the Antarctic soils analysed in this study (data extracted from [21] - Severgnini, M.; Canini, F.; Consolandi, C.; Camboni, T.; Paolo D'Acqui, L.; Mascalchi, C.; Ventura, S.; Zucconi, L. Highly Differentiated Soil Bacterial Communities in Victoria Land Macro-Areas (Antarctica). *FEMS Microbiol. Ecol.* **2021**, *97*, fiab087. https://doi.org/10.1093/femsec/fiab087).

	N %	C%	C/N ratio	pH	Na (cmol/kg)	K (cmol/kg)	Mg (cmol/kg)	Ca (cmol/kg)	CEC (cmol/kg)	Soil Moisture %	Sand %	Coarse silt %	Fine silt %	Clay %
**Ap.I 1**	0.202	5.87	29.1	7.76	0.06	0.14	0.4	0.25	0.85	0.595	74.1	12.3	12.9	0.7
**Ap.I 2**	0.243	6.31	26											
**B.By 1**	0.085	2.07	24.4	8.32	0.07	0.33	1.44	2.43	3.93	0.71	70.4	4.1	18.4	7.2
**B.By 2**	0.035	0.88	25.14											
**Cp.K 1**	0.103	0.91	8.8	7.6	0.14	0.34	2.18	1.89	6.42	0.432	93.7	2.5	3	0.8
**Cp.K 2**	0.066	1.87	28.33							0.35				
**Ed.P 2**	0.028	0.565	2.54	6.595	0.42	0.29	0.22	0.37	1.29	0.176	81	8.6	9.6	0.9
**Ed.P 3**	0.004	0.05	12.5	6.61	0.78	0.47	0.2	0.41	1.85	0.18	95.8	3.3	0.8	0.2
**Ed.P 4**	0.009	0.03	3.3	6.79	0.37	0.45	0.24	0.01	1.07	0.18	97.8	1.8	0.2	
**Ky.I 1**	0.209	6.37	30.5	7.3	0.55	0.42	0.69	0.54	4.91	14.2	75.4	12.2	11.7	0.7
**Ky.I 2**	0.227	6.44	28.4											
**Pr.I 1**	1.093	9.04	8.27	7.1	0.87	0.22	1.87	2.75	0.17	1.81	49.92	19	28.15	2.94
**Pr.I 2**	0.868	6.62	7.63							0.99				
**Lk.F 1.1**	0.019	0.33	17.4	7.81	2.12	0.45	1.15	2.06	3.53	0.18	87.2	3.2	6.3	3.3
**Lk.F 1.2**	0.01	0.03	3							0.473				
**Lk.F 1.3**	0.009	0.05	5.6							0.181				
**Lk.H 1.1**	0.008	0.11	13.8	7.98	0.07	0.36	0.88	0.84	2.16	0.088	93.4	2.7	3	1
**Lk.H 1.2**	0.0125	0.135	10.8							0.0895				
**Lk.J 1.1**	0.019	0.02	1.1	6.8	8.32	0.4	1.5	0.75	10.97	0.178	87.9	1.3	5.5	5.4
**Lk.J 1.2**	0.005	0.144	31.7	6.56	1.59	0.84	2.23	1.39	6.05	0.348	90.2	0.6	4.4	4.8

Table S2. Peak picking (chemical shift - ppm) and the corresponding peak intensities from ^{1}H-NMR spectra of extracts of Antarctic soils (Edmonson Point site 2) obtained by different extraction solvents: **A**) DMSO; **B**) H_2O/MeOH (1:1), and **C**) H_2O/MeCN (7:3). Samples were dissolved at final concentration of 15mg/mL in d_6-DMSO (**A**) and in 10 mM phosphate buffer (PB) in D_2O, pH 7.2 (**B** and **C**), with 0.5 mM TSP.

A) Extract in DMSO

	ppm	Intensity		ppm	Intensity
1	11.20	9587.7	21	2.61	66108.2
2	11.16	22425.4	22	2.57	11003.8
3	10.10	34779.8	23	2.55	3613277.1
4	9.21	13846.3	24	2.55	3445335.7
5	5.33	26736.5	25	2.51	1019956.1
6	5.18	14800.4	26	2.51	1049092.7
7	4.91	13703.4	27	2.43	26964.4
8	4.52	404886.9	28	2.39	18667.1
9	4.50	337998.6	29	2.34	78714.4
10	3.83	38107.4	30	2.34	76497.7
11	3.78	25219.8	31	2.02	29817.8
12	3.65	38621.7	32	1.97	24769.5
13	3.58	69298.2	33	1.46	18511.3
14	3.43	12643241.2	34	1.23	201231.9
15	3.25	67249.5	35	0.85	54955.9
16	3.11	42179.0	36	0.84	35204.9
17	2.99	19740.2	37	-0.02	108957.9
18	2.95	18507.1	38	-0.02	107391.9
19	2.89	15705.1	39	-0.07	50811.7
20	2.66	18724.4			

B) **Extract in H_2O/MeOH (1:1)**

	ppm	Intensity		ppm	Intensity		ppm	Intensity		ppm	Intensity		ppm	Intensity
1	8.46	51440.3	29	4.08	101194.0	57	3.69	351973.5	85	3.27	144877.1	113	2.05	53543.0
2	7.80	13443.0	30	4.07	124461.8	58	3.68	328503.4	86	3.26	149308.4	114	1.92	802985.4
3	7.43	15530.9	31	4.04	451400.3	59	3.67	334218.6	87	3.25	189643.8	115	1.91	66752.0
4	7.34	17883.8	32	4.02	464642.7	60	3.66	306297.0	88	3.23	128131.7	116	1.89	47364.0
5	6.91	21447.7	33	4.00	425342.1	61	3.65	140309.7	89	3.21	400527.3	117	1.55	63335.4
6	5.45	45882.4	34	3.97	148174.8	62	3.64	135520.7	90	3.13	189769.5	118	1.49	297026.2
7	5.41	57631.1	35	3.95	192246.7	63	3.63	89379.3	91	3.03	36154.3	119	1.48	294534.6

8	5.24	127509.9	36	3.94	207773.4	64	3.61	156905.6	92	3.02	54753.1	120	1.34	176357.0
9	5.23	131706.3	37	3.91	463480.6	65	3.59	354193.7	93	3.01	39728.7	121	1.32	200918.7
10	5.20	25506.0	38	3.91	422637.0	66	3.58	749761.9	94	2.81	21289.9	122	1.07	43908.2
11	5.03	49254.1	39	3.89	526814.2	67	3.57	390751.7	95	2.75	38972.7	123	1.06	70115.4
12	5.01	135206.8	40	3.89	677901.6	68	3.56	575194.0	96	2.73	189096.5	124	1.05	97251.0
13	4.66	81870.4	41	3.87	274009.6	69	3.54	194066.2	97	2.68	42406.9	125	1.04	98490.3
14	4.64	86334.9	42	3.85	289310.7	70	3.53	156981.9	98	2.66	25348.3	126	1.02	67044.7
15	4.29	49350.5	43	3.84	449947.4	71	3.53	179507.5	99	2.57	45428.6	127	1.01	64003.0
16	4.25	120277.1	44	3.82	424697.9	72	3.51	168317.9	100	2.54	35296.2	128	1.00	106806.6
17	4.24	110975.3	45	3.82	417394.4	73	3.49	288549.7	101	2.41	164889.4	129	0.99	107954.4
18	4.23	66451.2	46	3.81	715983.1	74	3.49	139011.5	102	2.39	36461.0	130	0.98	108624.2
19	4.22	68372.1	47	3.79	675202.1	75	3.48	232084.4	103	2.37	72169.0	131	0.97	172951.0
20	4.19	48404.4	48	3.78	391157.4	76	3.47	147863.4	104	2.36	71267.3	132	0.96	137115.7
21	4.17	69930.7	49	3.77	490907.1	77	3.46	135728.1	105	2.35	57081.1	133	0.94	97731.6
22	4.16	124680.0	50	3.76	498605.7	78	3.45	212614.2	106	2.31	52425.1	134	0.93	73225.6
23	4.15	155020.9	51	3.75	439533.4	79	3.44	190093.4	107	2.30	88457.7	135	0.91	68201.7
24	4.13	222473.2	52	3.74	434494.2	80	3.43	123390.1	108	2.29	60318.8	136	0.88	92735.8
25	4.13	170962.3	53	3.73	421290.7	81	3.42	245813.2	109	2.19	65053.3	137	0.07	134037.2
26	4.12	275922.0	54	3.72	925612.0	82	3.41	140702.8	110	2.17	81205.8	138	-0.00	1438756.8
27	4.11	303613.2	55	3.71	345560.0	83	3.40	205646.8	111	2.08	50906.1			
28	4.10	87891.3	56	3.70	835031.3	84	3.39	82366.6	112	2.06	59509.8			

C) Extract in H_2O/MeCN (7:3)

	ppm	Intensity		ppm	Intensity		ppm	Intensity		ppm	Intensity		ppm	Intensity		ppm	Intensity		ppm	Intensity
1	8.46	50610.4	29	4.27	81580.0	57	3.96	136573.4	85	3.67	384513.6	113	3.41	231291.4	141	2.41	203660.0	169	1.55	55835.5
2	7.81	20547.2	30	4.26	82106.1	58	3.95	191953.8	86	3.67	277847.3	114	3.40	75389.3	142	2.39	31101.6	170	1.49	346385.9
3	7.80	20422.3	31	4.25	125974.1	59	3.94	224191.1	87	3.66	340688.1	115	3.39	97516.4	143	2.37	74812.1	171	1.48	342652.7
4	7.43	15497.9	32	4.24	111560.9	60	3.93	189906.8	88	3.65	154324.8	116	3.35	16337.6	144	2.37	47477.1	172	1.37	44376.5
5	7.42	11907.9	33	4.23	77851.5	61	3.91	549630.3	89	3.64	128489.5	117	3.30	22380.5	145	2.36	73448.9	173	1.34	405440.9
6	7.34	18307.5	34	4.22	85490.3	62	3.91	465256.0	90	3.63	92222.0	118	3.28	33721.2	146	2.35	61810.7	174	1.33	428832.5
7	7.33	15700.5	35	4.20	47201.1	63	3.89	597411.9	91	3.61	170097.2	119	3.27	161270.5	147	2.31	66142.3	175	1.21	30153.9
8	7.19	11244.9	36	4.19	63560.7	64	3.89	733553.3	92	3.59	369957.4	120	3.26	176459.5	148	2.30	122110.8	176	1.20	27184.2
9	6.93	18482.9	37	4.17	77493.4	65	3.87	275825.5	93	3.58	895058.5	121	3.25	213942.4	149	2.29	74961.8	177	1.07	43796.0
10	6.91	25462.6	38	4.16	132448.5	66	3.86	277907.9	94	3.57	432328.8	122	3.23	155039.5	150	2.24	75184.2	178	1.06	72256.4
11	6.52	9541.9	39	4.15	160190.9	67	3.85	306933.9	95	3.56	674250.1	123	3.21	372034.2	151	2.19	66217.4	179	1.05	106281.0

12	5.91	9738.1	40	4.13	267728.6	68	3.84	465836.6	96	3.55	205203.9	124	3.19	21846.8	152	2.18	83267.5	180	1.05	48297.4
13	5.45	44108.1	41	4.13	232391.6	69	3.82	452169.8	97	3.55	205012.4	125	3.13	109194.0	153	2.16	57683.3	181	1.04	106006.3
14	5.42	57789.1	42	4.12	310111.4	70	3.82	435664.8	98	3.54	189750.8	126	3.11	24146.2	154	2.14	45863.9	182	1.02	71298.9
15	5.25	23676.8	43	4.11	345768.0	71	3.81	863996.1	99	3.53	158109.2	127	3.03	57632.1	155	2.13	43335.7	183	1.01	67222.6
16	5.24	153725.0	44	4.11	122158.4	72	3.79	809133.3	100	3.53	195127.3	128	3.02	75479.8	156	2.12	42168.0	184	1.00	118431.7
17	5.23	152981.2	45	4.10	97903.6	73	3.78	471955.9	101	3.51	197941.6	129	3.01	54298.3	157	2.08	62065.7	185	0.99	121299.8
18	5.20	32191.9	46	4.08	115918.2	74	3.77	552899.9	102	3.49	342666.7	130	2.84	16278.7	158	2.06	61584.4	186	0.98	116099.0
19	5.20	32220.9	47	4.07	135061.1	75	3.76	527360.1	103	3.49	135528.5	131	2.81	23136.1	159	2.05	53461.4	187	0.97	190080.3
20	5.04	50035.5	48	4.06	109259.5	76	3.75	443997.1	104	3.48	256794.5	132	2.80	23905.1	160	2.02	31940.8	188	0.96	143820.0
21	5.03	53339.3	49	4.04	485924.1	77	3.74	454134.6	105	3.47	141423.1	133	2.75	42392.8	161	2.01	37817.9	189	0.94	100433.4
22	5.02	113810.2	50	4.02	487871.9	78	3.73	444241.0	106	3.47	147047.2	134	2.72	21892.3	162	1.92	1223068.9	190	0.93	69857.4
23	5.01	146966.8	51	4.01	128996.2	79	3.72	1116757.7	107	3.47	146219.8	135	2.70	34761.0	163	1.91	80859.6	191	0.92	57219.5
24	4.97	17817.8	52	4.00	426655.4	80	3.71	376082.6	108	3.46	220904.7	136	2.68	32339.5	164	1.89	55852.9	192	0.91	54953.9
25	4.66	12541.4	53	4.00	460894.8	81	3.70	1076639.0	109	3.44	183741.4	137	2.67	25535.3	165	1.88	30784.3	193	0.88	77458.6
26	4.64	17326.3	54	4.00	418471.9	82	3.70	524108.2	110	3.43	132919.7	138	2.65	22107.6	166	1.73	32429.5	194	-0.00	1125706.5
27	4.30	50839.9	55	3.98	123196.6	83	3.69	362244.0	111	3.42	290503.4	139	2.57	23195.8	167	1.72	31875.4			
28	4.29	51880.6	56	3.97	148853.1	84	3.68	360650.4	112	3.41	148886.5	140	2.44	23971.8	168	1.70	31870.5			

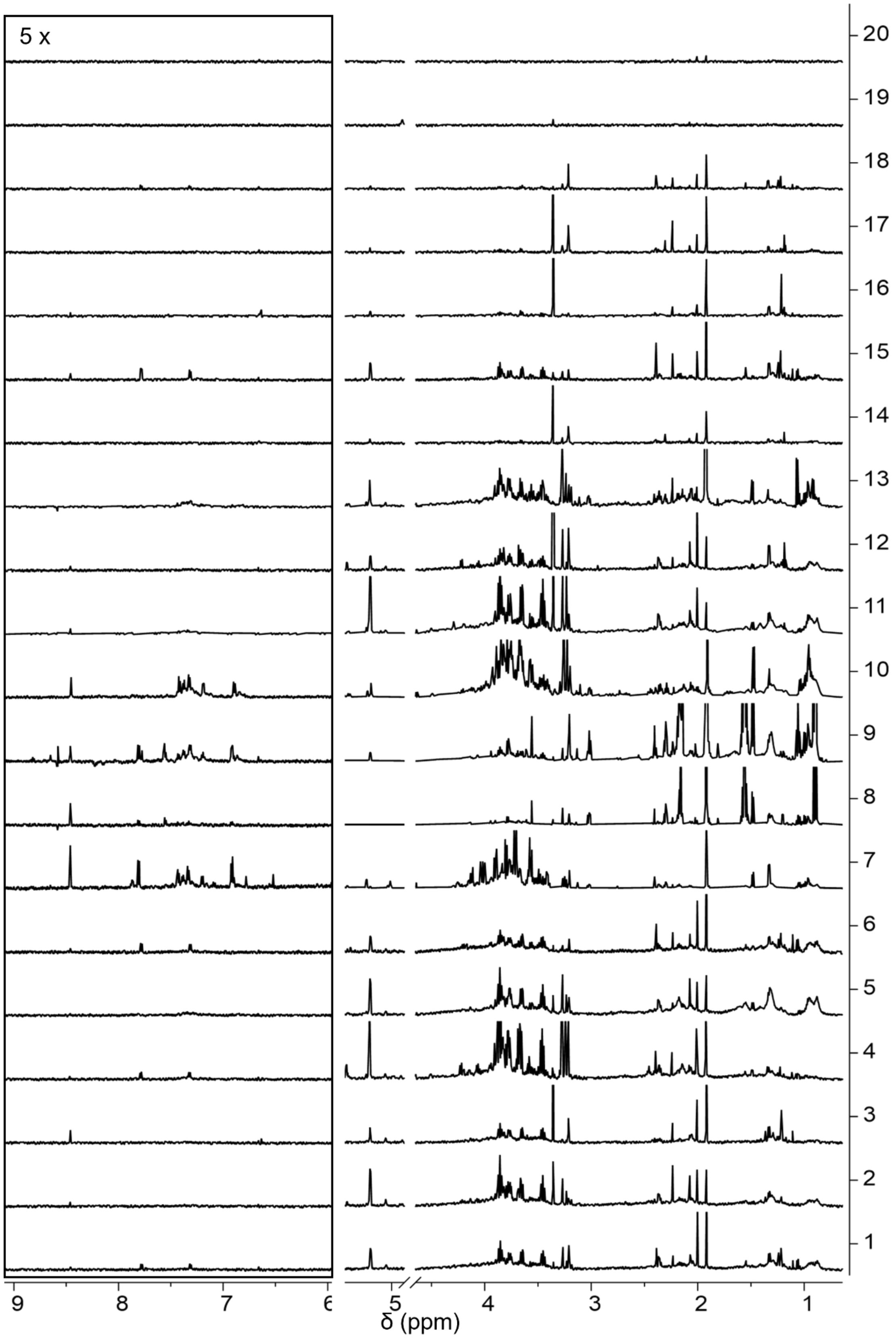

Figure S1. ^{1}H-NMR spectra of extracts of Antarctic soils from different sites obtained in H_2O/MeCN (7:3). Samples are dissolved at final concentration of 15mg/mL in 10 mM PB in D_2O, pH 7.2, with 0.5 mM TSP. 1) Ap.I 1; 2) Ap.I 2; 3) B.By 1; 4) B.By 2; 5) Cp.K 1; 6)Cp.K 2; 7) Ed.P 2; 8) Ed.P 3; 9) Ed.P 4; 10) Ky.I 1; 11) Ky.I 2; 12) Pr.I 1; 13) Pr.I 2; 14) Lk.F 1.1; 15) Lk.F 1.2; 16) Lk.F 1.3; 17) Lk.H 1.1; 18) Lk.H 1.2; 19) Lk.J 1.1; 20) Lk.J 1.2.

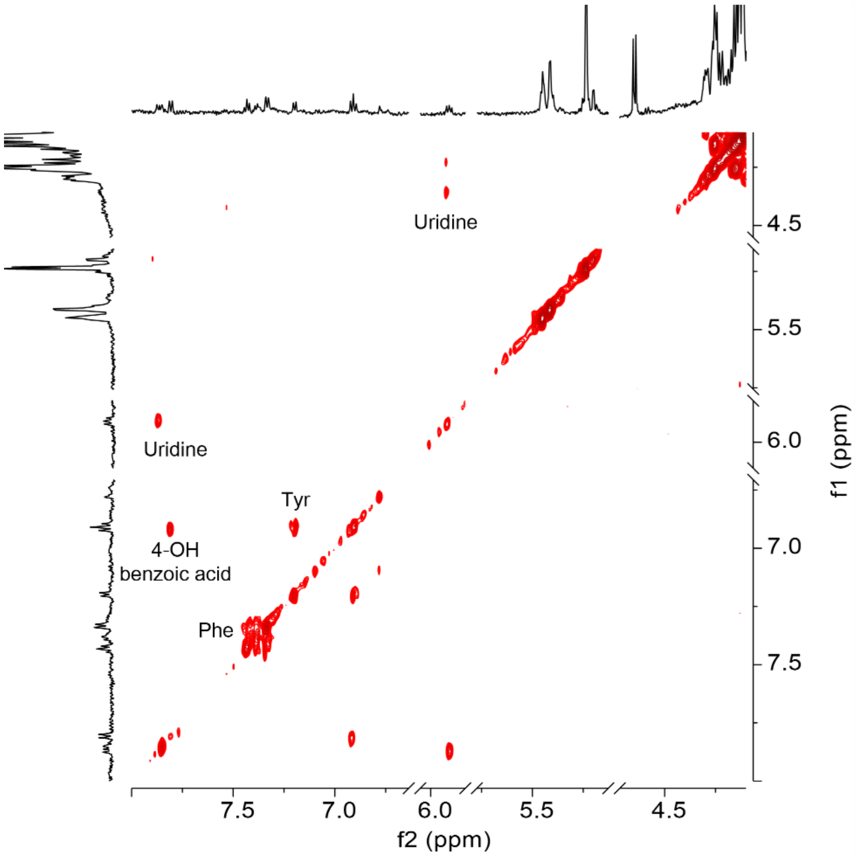

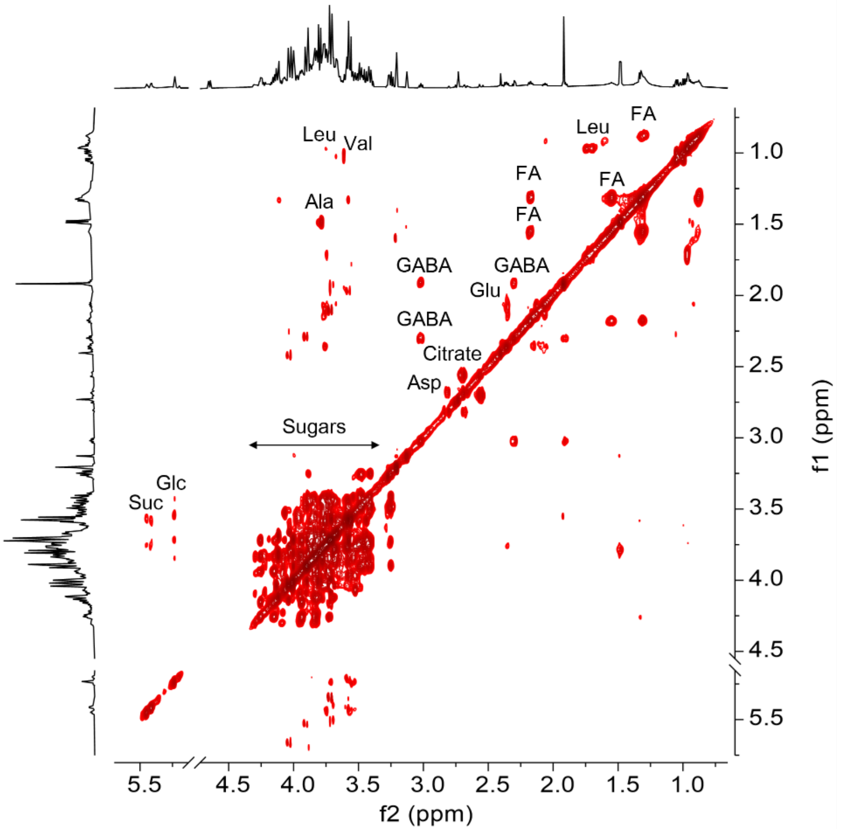

Figure S2. Expansions of two regions of ^{1}H,^{1}H-TOCSY spectrum of Antarctic soil extract Ed.P 2 in 10 mM PB in D_2O. The corresponding ^{1}H-NMR spectra are reported along the axes. The resonances of some of the most important metabolites are indicated on the 2D spectra.

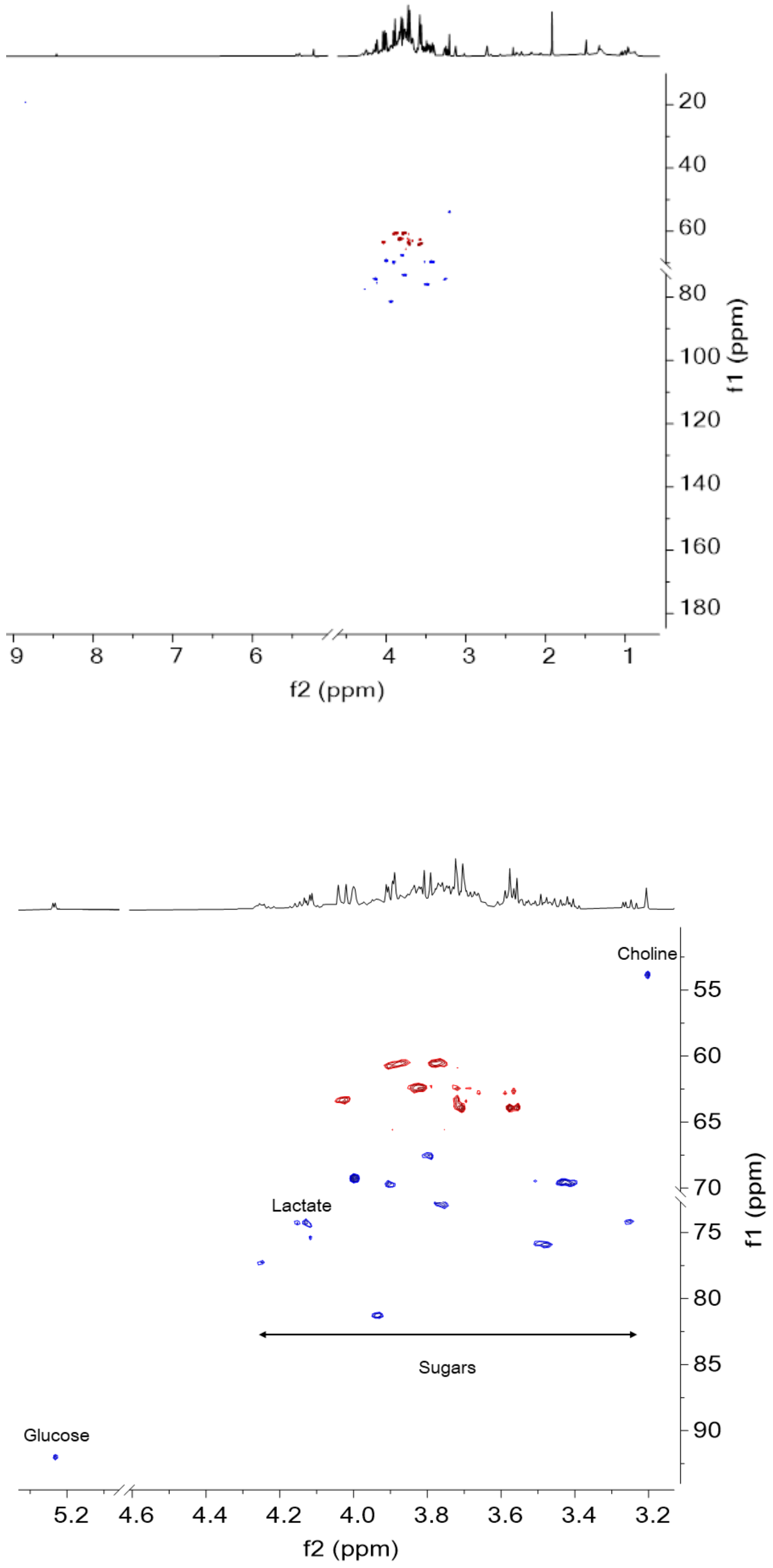

Figure S3. $^{1}H,^{13}C$-HSQC spectrum of Antarctic soil extract Ed.P 2 in 10 mM PB in D_2O. The corresponding ^{1}H-NMR spectrum is reported.

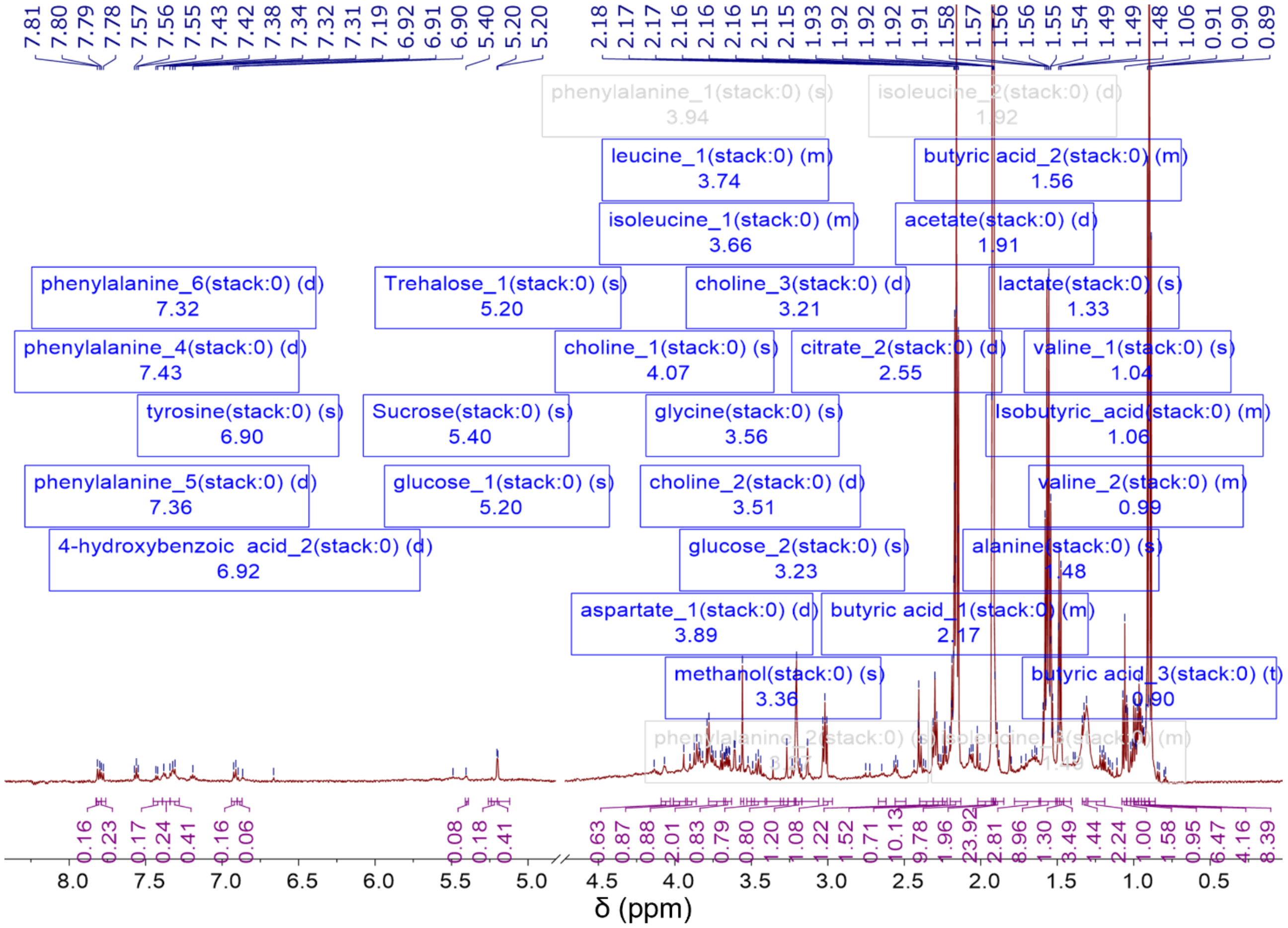

Figure S4. Output of SMA plug-in of MestreNova software on a spectrum of Antarctic soil extract Ed.P 4 in 10 mM PB in D_2O. Labels with the name of the molecule, the chemical shift and multiplicity are automatically reported for each signal.

MS/MS spectra of identified compounds

Experimental spectra (ES-) reported in comparison with matched reference spectra.

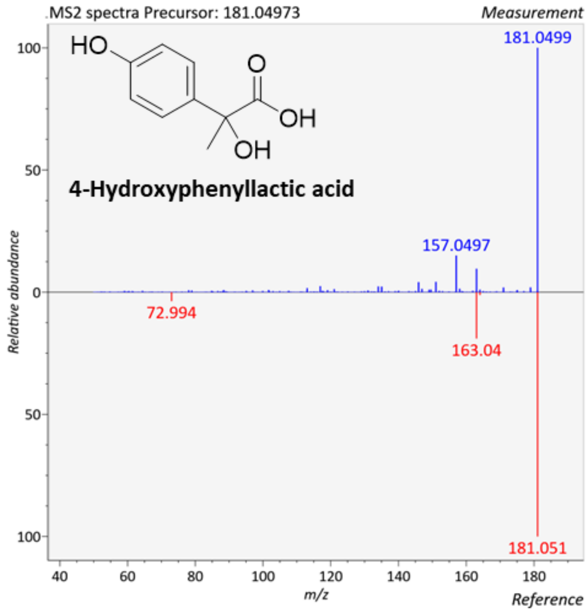

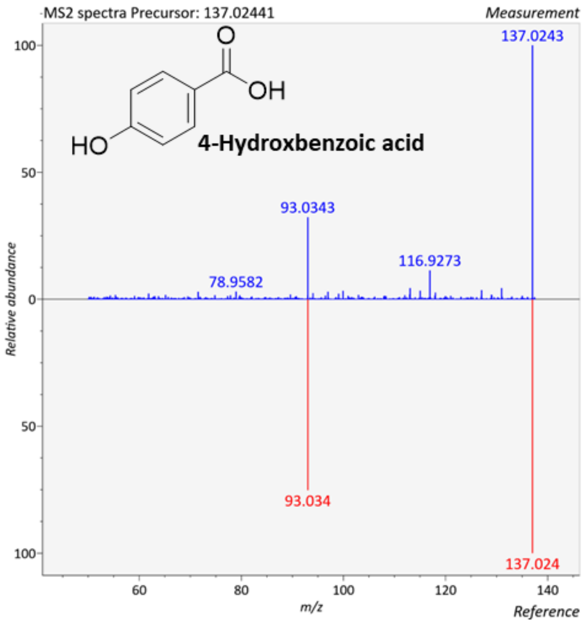

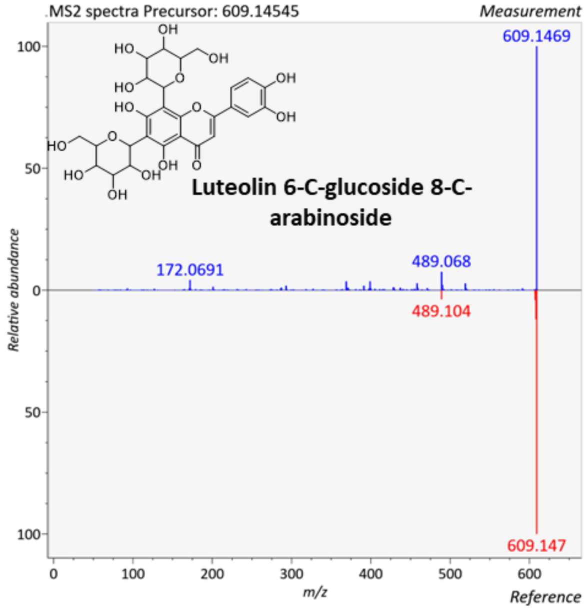

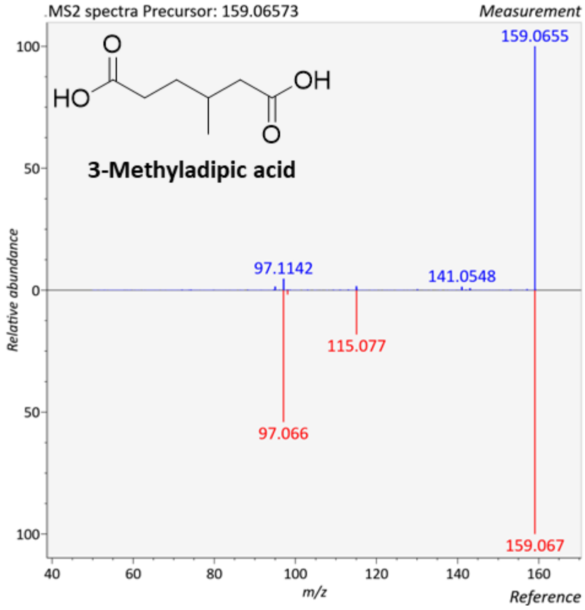

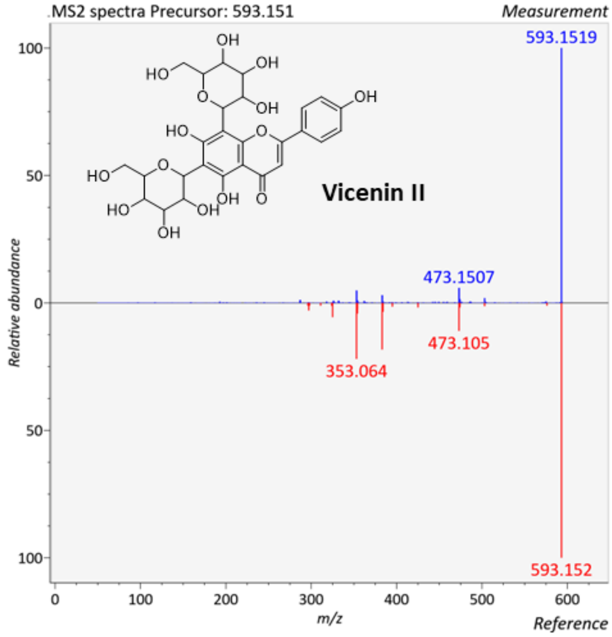
MS2 spectra Precursor: 593.151
Measurement
Reference
Vicenin II
593.1519
473.1507
353.064
473.105
593.152
Relative abundance
m/z

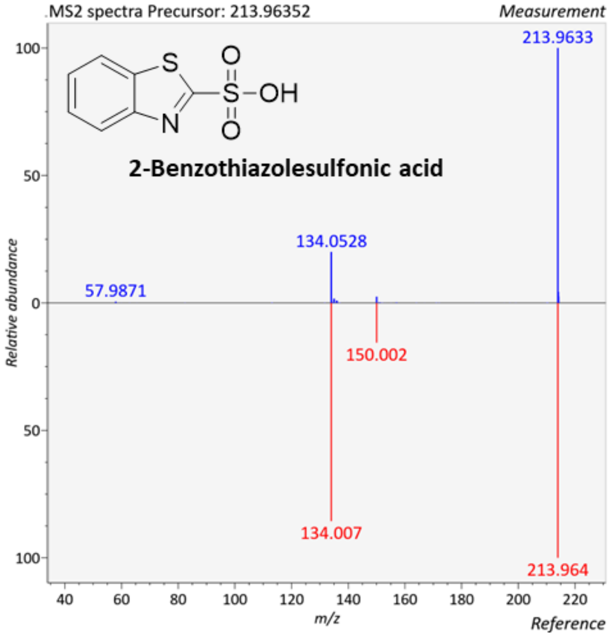
MS2 spectra Precursor: 213.96352
Measurement
Reference
2-Benzothiazolesulfonic acid
213.9633
134.0528
57.9871
150.002
134.007
213.964
Relative abundance
m/z

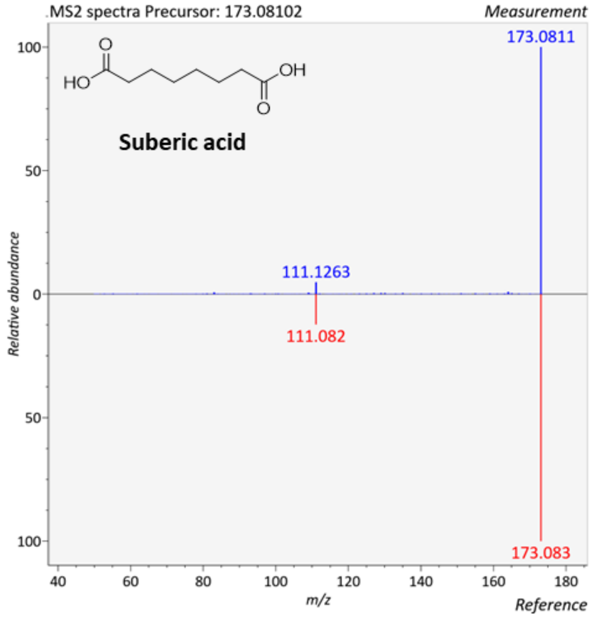
MS2 spectra Precursor: 173.08102
Measurement
Reference
Suberic acid
173.0811
111.1263
111.082
173.083
Relative abundance
m/z

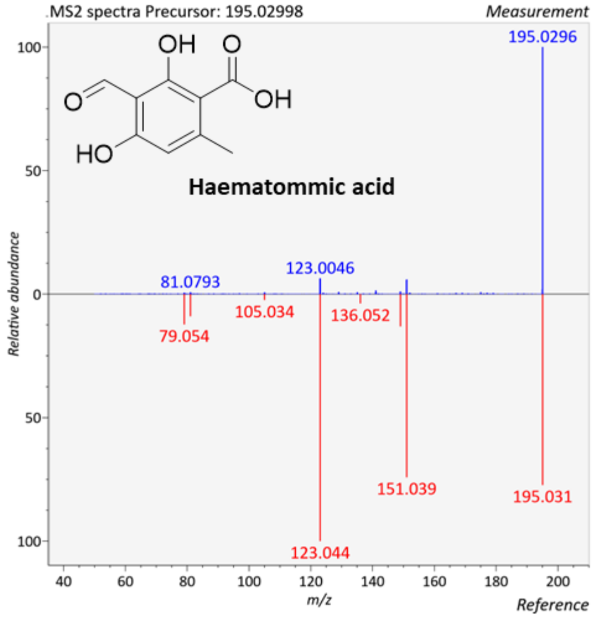
MS2 spectra Precursor: 195.02998
Measurement
Reference
Haematommic acid
195.0296
123.0046
81.0793
105.034
136.052
79.054
151.039
195.031
123.044
Relative abundance
m/z

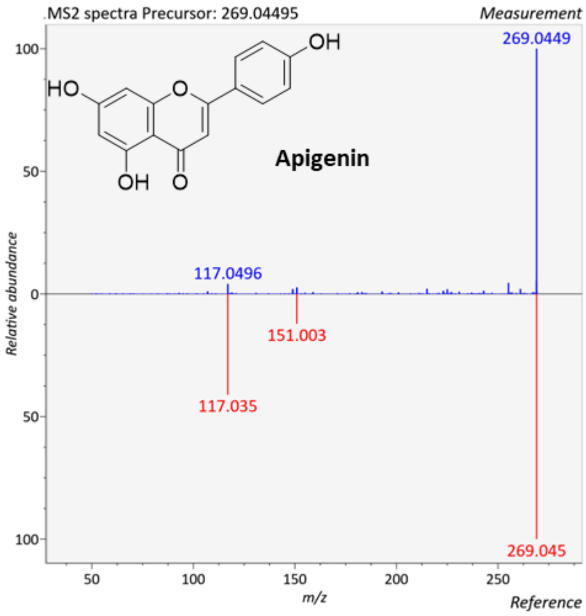
MS2 spectra Precursor: 269.04495
Measurement
Apigenin
269.0449
117.0496
151.003
117.035
269.045
Relative abundance
m/z
Reference

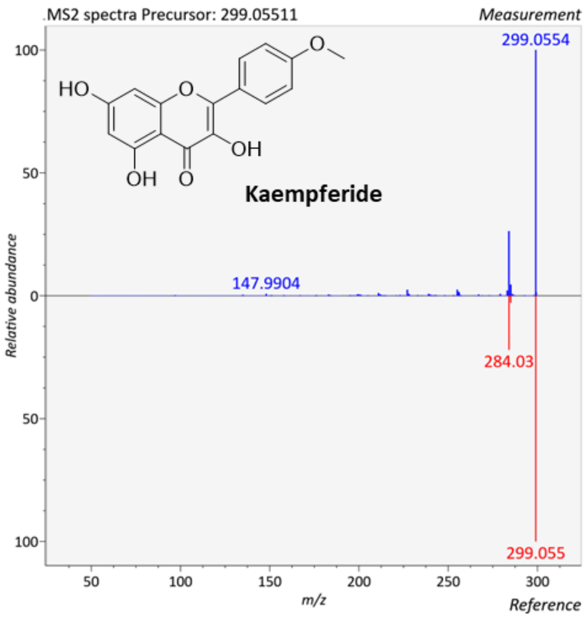
MS2 spectra Precursor: 299.05511
Measurement
Kaempferide
299.0554
147.9904
284.03
299.055
Relative abundance
m/z
Reference

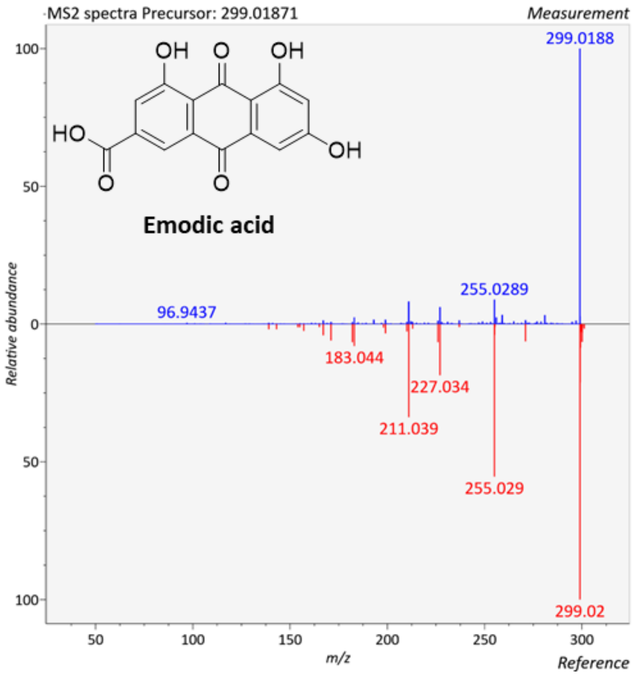
MS2 spectra Precursor: 299.01871
Measurement
Emodic acid
299.0188
96.9437
255.0289
183.044
227.034
211.039
255.029
299.02
Relative abundance
m/z
Reference

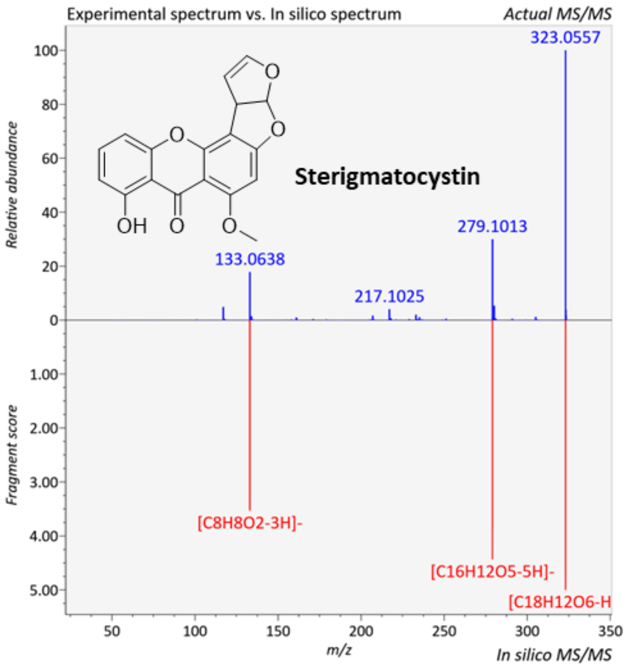
Experimental spectrum vs. In silico spectrum
Actual MS/MS
Sterigmatocystin
323.0557
279.1013
133.0638
217.1025
[C8H8O2-3H]-
[C16H12O5-5H]-
[C18H12O6-H
Relative abundance
Fragment score
m/z
In silico MS/MS

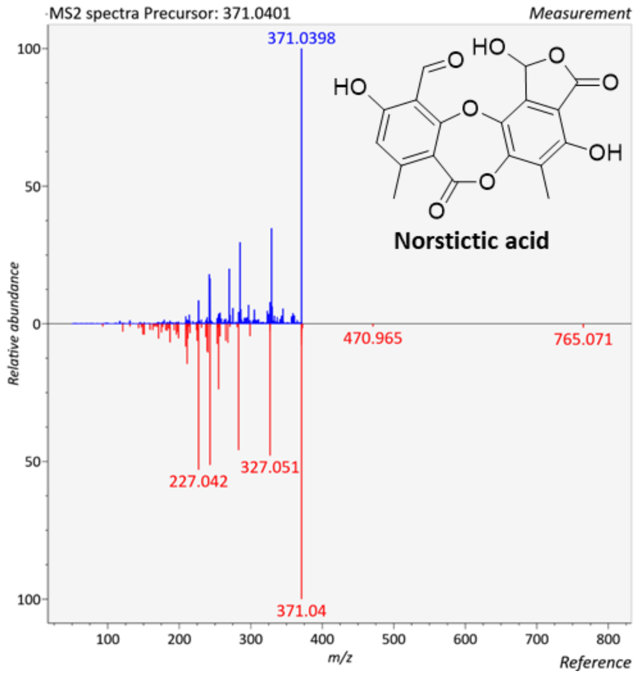
MS2 spectra Precursor: 371.0401
Measurement
371.0398
Norstictic acid
470.965
765.071
227.042
327.051
371.04
Relative abundance
m/z
Reference

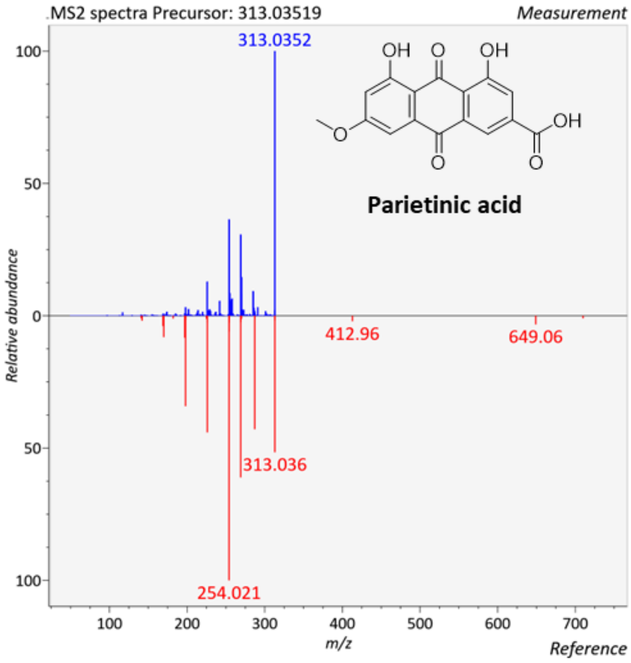
MS2 spectra Precursor: 313.03519
Measurement
313.0352
Parietinic acid
412.96
649.06
313.036
254.021
Relative abundance
m/z
Reference

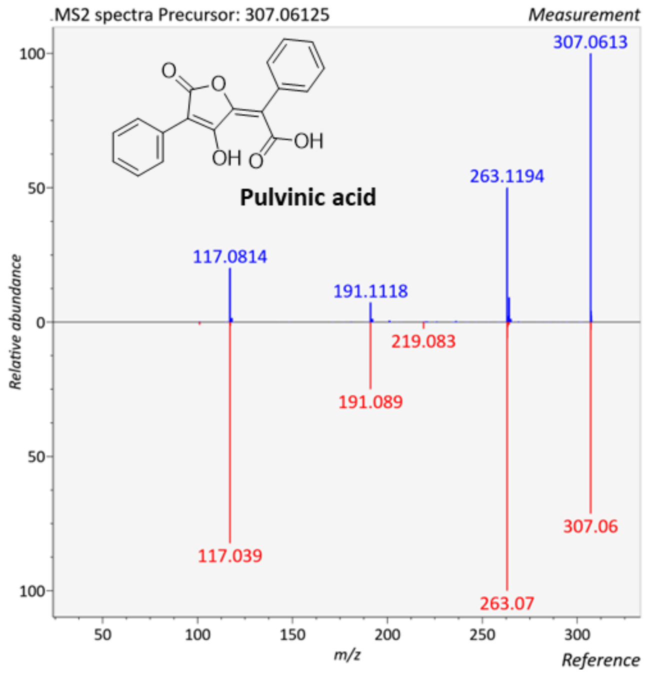
MS2 spectra Precursor: 307.06125
Measurement
307.0613
Pulvinic acid
263.1194
117.0814
191.1118
219.083
191.089
307.06
117.039
263.07
Relative abundance
m/z
Reference

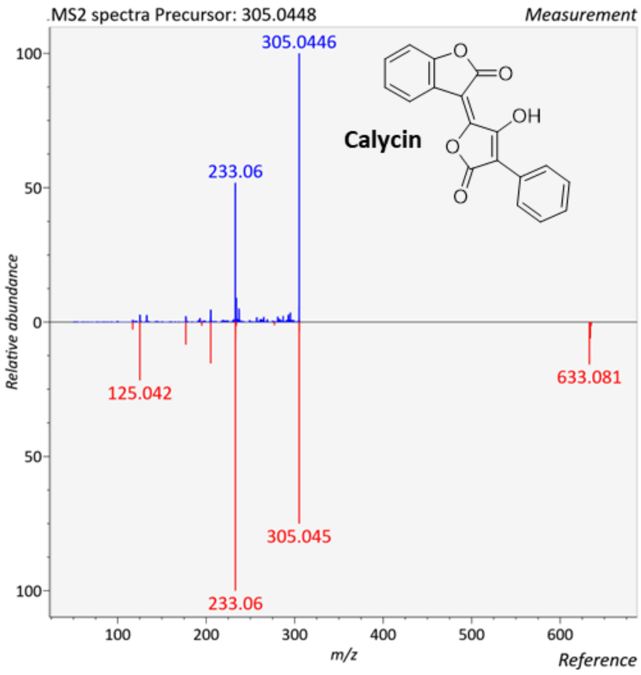
MS2 spectra Precursor: 305.0448
Measurement
305.0446
Calycin
233.06
125.042
633.081
305.045
233.06
Relative abundance
m/z
Reference

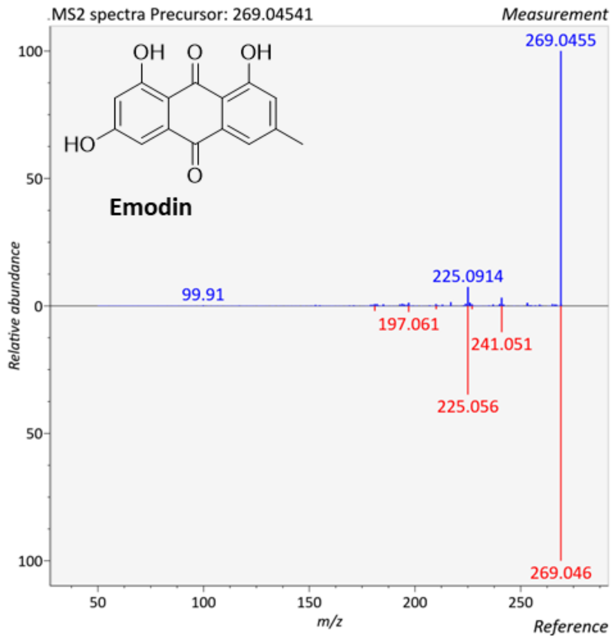
MS2 spectra Precursor: 269.04541
Measurement
Emodin
269.0455
225.0914
99.91
197.061
241.051
225.056
269.046
Relative abundance
m/z
Reference

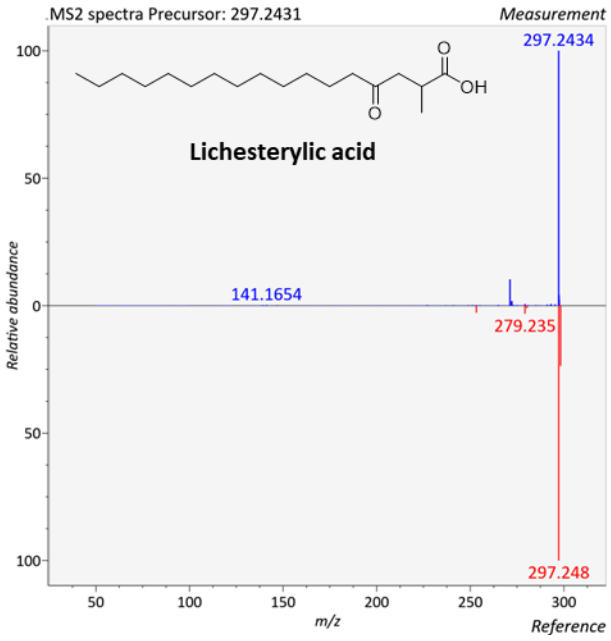
MS2 spectra Precursor: 297.2431
Measurement
Lichesterylic acid
297.2434
141.1654
279.235
297.248
Relative abundance
m/z
Reference

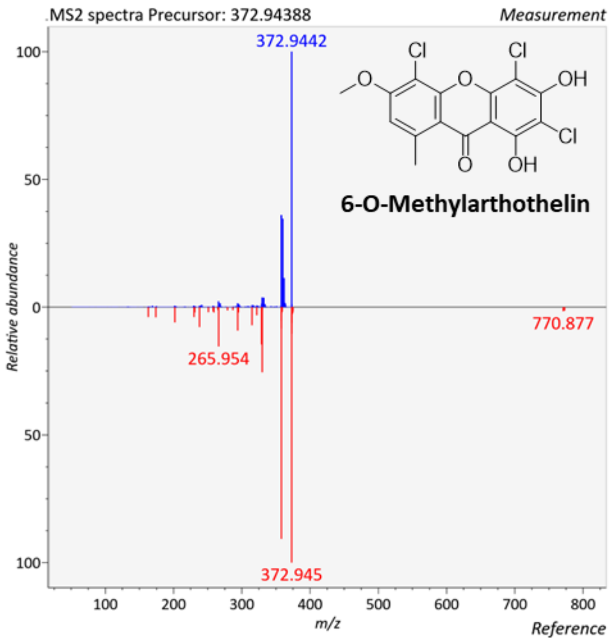
MS2 spectra Precursor: 372.94388
Measurement
6-O-Methylarthothelin
372.9442
770.877
265.954
372.945
Relative abundance
m/z
Reference

Experimental spectra (ES+) reported in comparison with matched reference spectra.

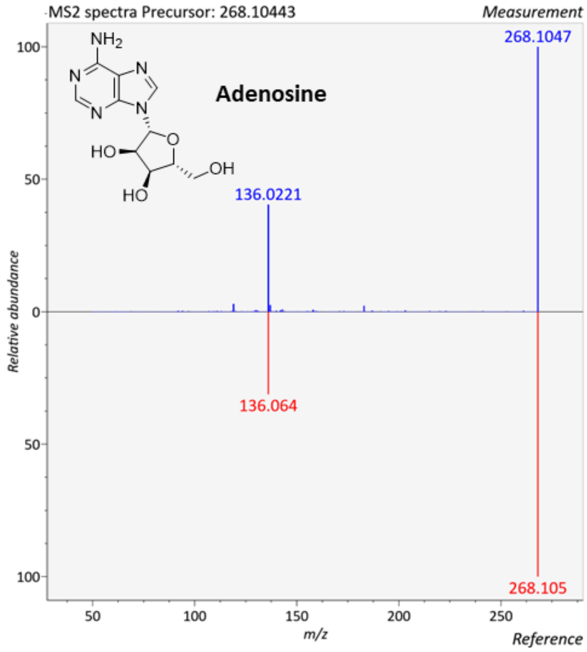

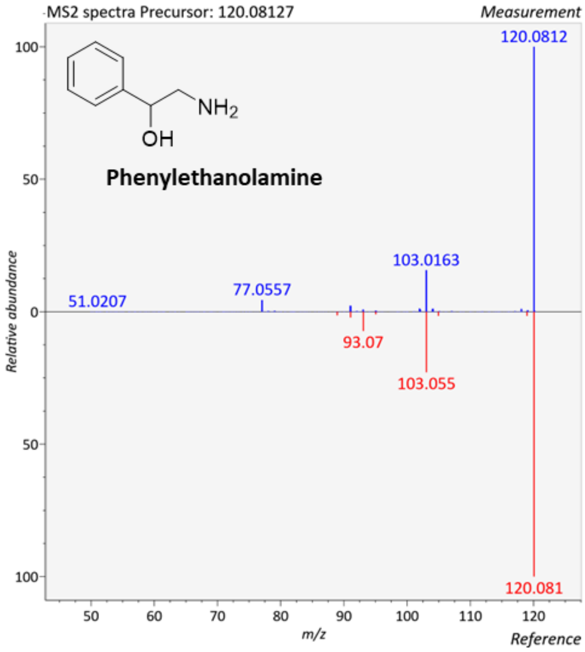

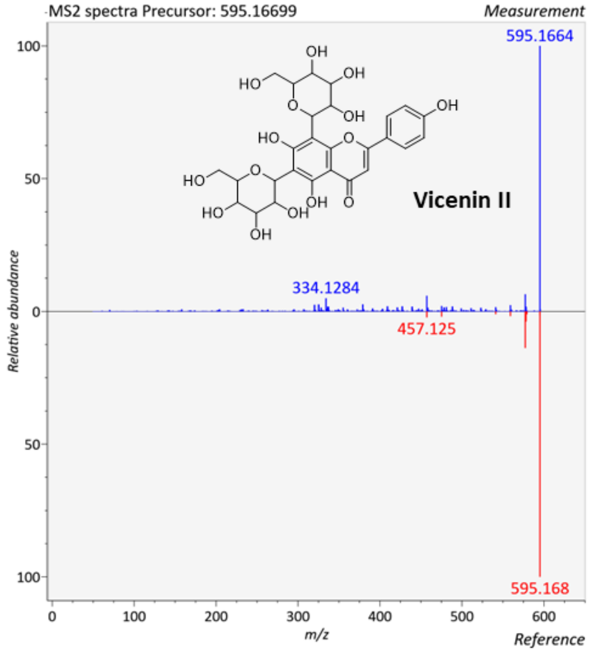

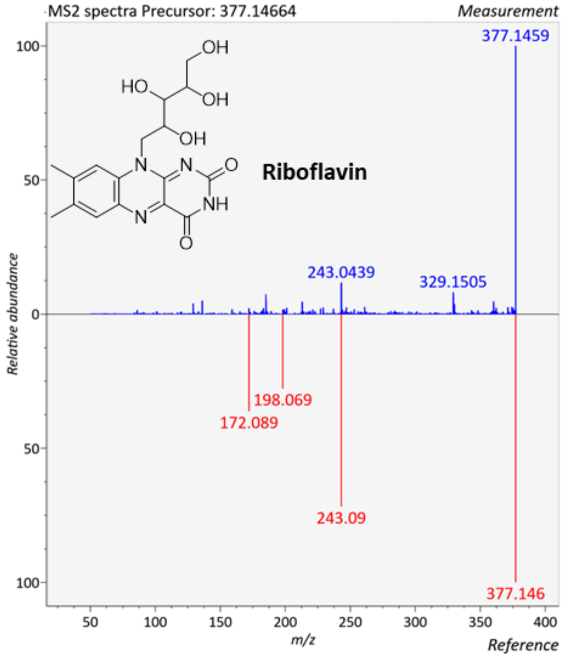

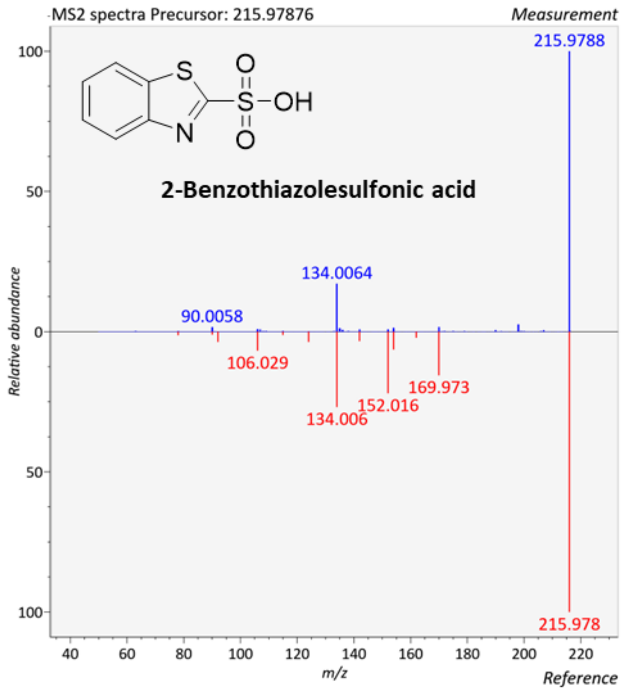
MS2 spectra Precursor: 215.97876
Measurement
2-Benzothiazolesulfonic acid
215.9788
134.0064
90.0058
106.029
134.006
152.016
169.973
215.978
Relative abundance
m/z
Reference

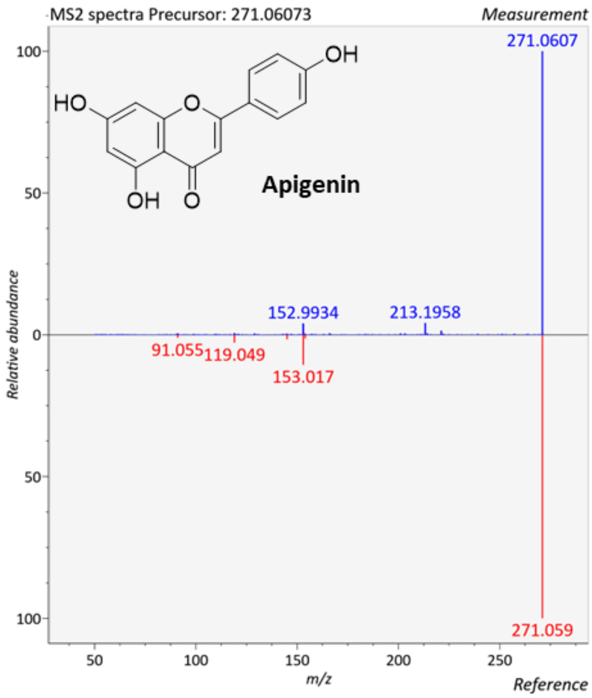
MS2 spectra Precursor: 271.06073
Measurement
Apigenin
271.0607
152.9934
213.1958
91.055
119.049
153.017
271.059
Relative abundance
m/z
Reference

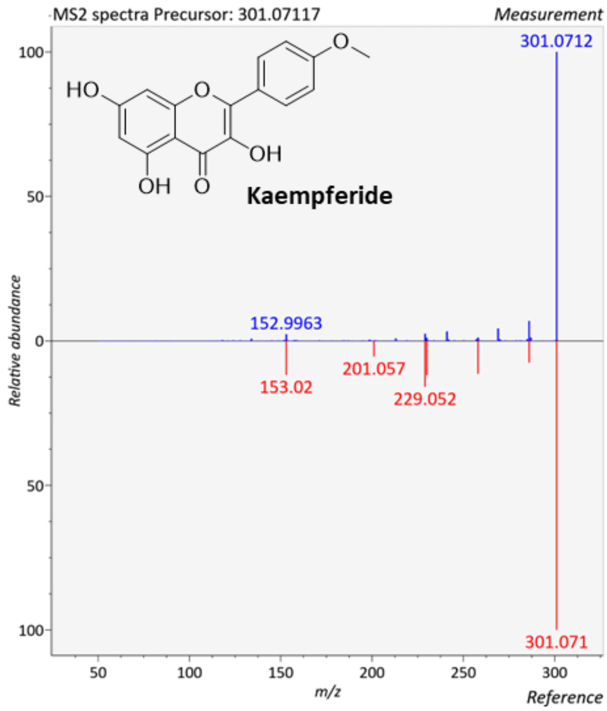
MS2 spectra Precursor: 301.07117
Measurement
Kaempferide
301.0712
152.9963
153.02
201.057
229.052
301.071
Relative abundance
m/z
Reference

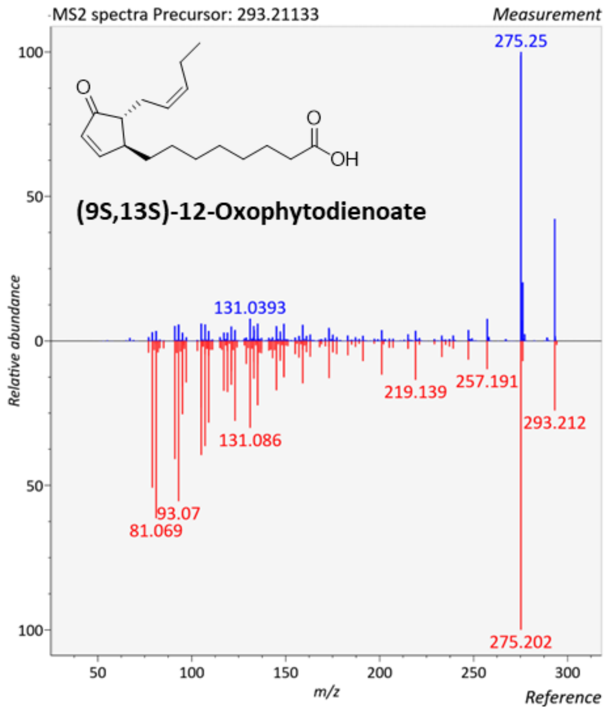
MS2 spectra Precursor: 293.21133
Measurement
(9S,13S)-12-Oxophytodienoate
275.25
131.0393
219.139
257.191
293.212
131.086
93.07
81.069
275.202
Relative abundance
m/z
Reference

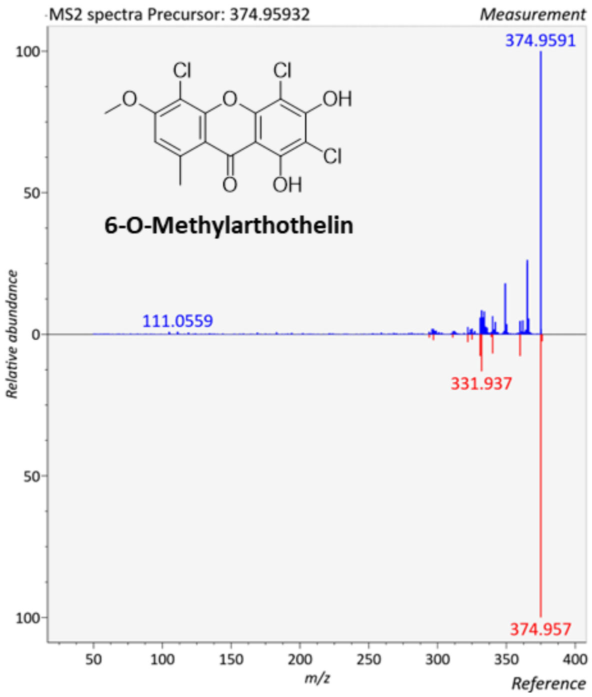
MS2 spectra Precursor: 374.95932
Measurement
374.9591
6-O-Methylarthothelin
111.0559
331.937
374.957
Relative abundance
m/z
Reference
